# Clinically Inspired Multimodal Treatment Using Induced Neural Stem Cells‐Derived Exosomes Promotes Recovery of Traumatic Brain Injury through Microglial Modulation

**DOI:** 10.1002/advs.202508574

**Published:** 2025-09-24

**Authors:** Jiaojiao Li, Maoxiang Xu, Boyu Cai, Xiangyu Li, Zhanping Liang, Xiaohuan Xia, Haitao Zhang, Zhiwen Zhang, Fei Tan, Jialin Charlie Zheng

**Affiliations:** ^1^ Department of ORL‐HNS Shanghai Fourth People's Hospital Affiliated to Tongji University School of Medicine Shanghai 200434 China; ^2^ Plasma Medicine and Surgical Implants Center Tongji University School of Medicine Shanghai 200092 China; ^3^ Center for Translational Neurodegeneration and Regenerative Therapy Tongji Hospital Affiliated to Tongji University School of Medicine Shanghai 200092 China; ^4^ Shanghai Frontiers Science Center of Nanocatalytic Medicine Tongji University Shanghai 200331 China; ^5^ Department of Neurosurgery the Fourth Medical Center of PLA General Hospital Beijing 100048 China; ^6^ Department of Neurosurgery Shanghai Fourth People's Hospital Affiliated to Tongji University School of Medicine Shanghai 200434 China; ^7^ Department of ORL‐HNS The Royal College of Surgeons of England London WC2A3PE UK

**Keywords:** exosomes, induced neural stem cells, injectable hydrogel, targeted delivery, traumatic brain injury

## Abstract

Traumatic brain injury (TBI) poses serious physical, psychosocial, and economic threats to millions of patients globally each year. While current treatment options, primarily surgery and medication, vary with TBI severity, there is no universal therapeutic agent applicable in both surgical and medical contexts. In the present study, exosomes derived from induced neural stem cells (iNSC‐Exo) as a versatile therapeutic agent for TBI are investigated using a preclinical murine model. The iNSC‐Exo treatment is found to exert therapeutic effects by mediating anti‐neuroinflammation and neuroprotection, thereby promoting functional and cognitive recovery in TBI mice. Besides, two clinically inspired administration modalities are established for iNSC‐Exo: local delivery and systemic delivery. Their efficacy is enhanced via a novel injectable hydrogel and RVG targeting for systemic delivery, respectively. Finally, the unprecedented single‐cell characterization of mouse brain tissue, both pre‐ and post‐iNSC‐Exo treatment, confirms that microglia represent the predominant type of cells affected. Two microglial subpopulations (i.e., Microglia_Nrg3 and Microglia_Rarb) are identified with a reduced state of differentiation, and their connectivity with neurons is predicted through activation of the NRXN signaling pathway. Overall, these findings demonstrated that iNSC‐Exo offers a versatile and potent treatment platform with clinical potential for TBI management.

## Graphical abstract

This study explores the therapeutic potential of iNSC‐Exo via local and systemic delivery in TBI mice. It is found that iNSC‐Exo enhances NRXN signaling through Microglia_Nrg3 and Microglia_Rarb, effectively promoting functional and neurological recovery, suggesting a new therapeutic strategy for TBI.

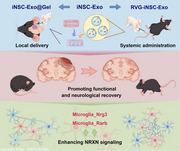

## 1 Introduction

Approximately 70 million patients suffer from traumatic brain injury (TBI) globally each year, which poses serious physical, psychosocial, and economic threats.^[^ ^1^ ^]^ Clinically, TBI severity can be graded as mild, moderate, and severe depending on the patient's Glasgow Coma Scale (GCS).^[^ ^2^ ^]^ Pathologically, TBI can be categorized as primary (e.g., axonal death, neuroinflammation, neurochemical change, and metabolic dysfunction) and secondary (e.g., ischemic and hypoxic damage, cerebral edema, raised intracranial pressure, hydrocephalus, and infection) injuries.^[^ ^3^ ^]^ Current treatment options for TBI are based on the severity of the injury, including medications, surgery, and rehabilitation, although none of these approaches can prevent primary injury and terminate the progression of secondary injury.^[^ ^4^ ^]^ Furthermore, the unique circumstances of each patient, such as the injury's location and severity, make medical and surgical treatment highly challenging.^[^ ^5^ ^]^ Therefore, there lack a “one‐size‐fits‐all” option for managing TBI. Over the past few decades, research has demonstrated that stem cells, especially neural stem cells (NSCs) and mesenchymal stem cells (MSCs), can mitigate the detrimental effects of post‐TBI.^[^ ^6^ ^]^

Exosomes, which are nanoscale, lipid bi‐layered extracellular vesicles, represent a potent alternative to traditional stem cell therapy given that they inherit their therapeutic effects from their parent cells (e.g., anti‐inflammation, immunomodulation, and tissue regeneration) without the limitations associated with their cellular counterparts.^[^ ^7^ ^]^ Exosomes derived from stem cells, for example, NSCs (NSC‐Exo), have demonstrated significant preclinical potential in treating various neurosurgical conditions, such as TBI.^[^ ^8^ ^]^ However, due to supply constraints, limited expandability in culture, and ethical considerations, NSCs may not be an ideal source for large‐scale manufacture of clinical‐grade exosomes. Our recent studies collected mouse fibroblast‐derived induced NSCs (iNSCs) through somatic cell reprogramming, opening a new window for obtaining exosomes derived from NSC‐like cells. Besides, iNSC‐derived exosomes (iNSC‐Exo) have demonstrated potential in the management of various neurological conditions, such as Alzheimer's disease^[^ ^9^ ^]^ and ischemic stroke.^[^ ^10^ ^]^ However, the efficacy of iNSC‐Exo in treating other neurosurgical disorders, such as TBI and spinal cord injury, remains largely unknown.

Most preclinical studies on stem cell‐derived exosomes (SC‐Exo) therapies for TBI^[^ ^8^, ^11^, ^12^, ^13^, ^14^, ^15^, ^16^ ^]^ employ over‐simplified systemic administration, for example, through tail‐vein injection, which might not reflect the diverse practicalities of clinical scenarios. Although ≈90% of TBI patients present with mild symptoms (GCS 13–15), the remaining 10% manifest moderate‐to‐severe manifestations often necessitating surgical interventions.^[^ ^17^ ^]^ Procedures like craniectomy performed for elevated intracranial pressure and hydrocephalus are often lifesaving. Leveraging craniectomy for local delivery of therapeutic agents, for example, exosomes, to the brain parenchyma could offer additional benefits over systemic administration as they reduce exosome accumulation in the liver and spleen while improving brain‐specific enrichment.^[^ ^18^ ^]^ Irrespective of the delivery modality, exosome modification or engineering can be performed to further enhance their therapeutic efficacy. These are exemplified by tagging intravenously infused exosomes with rabies viral glycoprotein (RVG) peptide for targeting the central nervous system (CNS),^[^ ^19^ ^]^ and achieving sustained exosome release from locally applied biomaterial post‐craniectomy.^[^ ^20^ ^]^

In this study, we present the first development of a “one‐size‐fits‐all” therapeutic platform based on iNSC‐Exo for managing TBI in a preclinical model. The primary objective of this study was to ascertain whether iNSC‐Exo could promote the recovery of TBI and reveal its therapeutic efficacy relative to NSC‐Exo. The secondary objective is to simulate various clinical treatment options in TBI by systemic administration and local delivery of iNSC‐Exo in a preclinical mouse model, with further optimization through RVG modification and injectable hydrogel, respectively. Finally, the cellular and molecular mechanisms of iNSC‐Exo therapy in TBI were explored using whole‐transcriptome amplification single‐cell RNA sequencing, highlighting the crosstalk among various subpopulations of microglia and neurons.

## 2 Results

### 2.1 Characterization of Neurospheres and Exosomes Demonstrates Strong Similarities between iNSC‐Exo and NSC‐Exo

**Figure 1:**
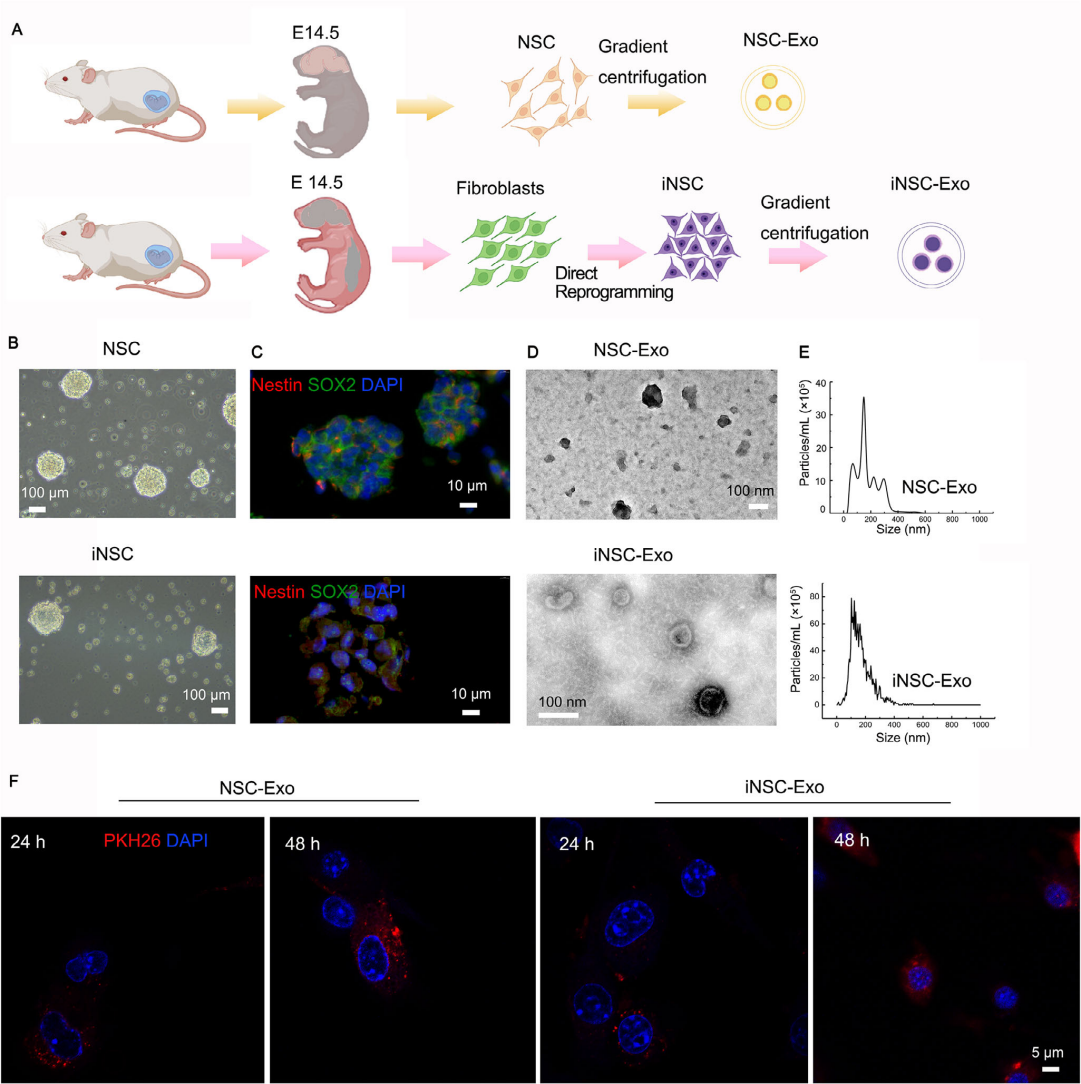
Characterization of neurospheres and exosomes demonstrates strong similarities between iNSC‐Exo and NSC‐Exo. A) Schematic of the extraction of NSC‐Exo and iNSC‐Exo. B) Photographs of NSC‐ and iNSC‐forming neurospheres after being cultured under proliferative conditions. C) Cells expressing SOX2‐ and Nestin‐specific immunoreactivities in the NSC and iNSC groups. D) TEM characterization of the morphology of NSC‐Exo and iNSC‐Exo. E) Particle size distribution of NSC‐Exo and iNSC‐Exo. F) Internalization of NSC‐Exo or iNSC‐Exo in BV2 cells. Labelling of exosomes and BV2 cell nuclei by PKH26 (red) and DAPI (blue), respectively.

To assess the therapeutic effects of exosomes on the recovery of TBI, we isolated NSC‐Exo and iNSC‐Exo for the preclinical study (**Figure** **1**A). In suspension culture, both NSC‐ and iNSC‐generated neurospheres exhibited a spherical morphology (Figure 1B). These neurospheres were positive for neural stem cell markers Nestin (red) and SOX2 (green) (Figure 1C). TEM revealed a cup‐shaped or elliptical morphology of exosomes with a diameter of less than 200 nm (Figure 1D). The particle size of the purified exosomes at 102 nm for NSC‐Exo and 150 nm for iNSC‐Exo, respectively, confirming the purification of exosomes (Figure 1E). Flow cytometry revealed strong expression of three specific protein markers of exosomes in the collected NSC‐Exo and iNSC‐Exo samples, including CD81, CD63, and CD9 (Figure S1, Supporting Information). The positive expression of PKH26 indicated that NSC‐Exo and iNSC‐Exo could be engulfed by both BV2 cells (Figure 1F) and PC12 cells (Figure S2, Supporting Information). Broadly distributed fluorescent spots were observed within the cytoplasm of almost all cells after 48 h of incubation. The results suggested that iNSC‐Exo and NSC‐Exo share strong similarities in their morphology, distribution, and cellular internalization. Furthermore, we investigated the dose‐dependent effects of iNSC‐Exo by treating cells with different concentrations (10, 20, and 30 µg per well). A significant positive correlation was observed between exosome dosage and exosomal uptake (Figure S3, Supporting Information).

### 2.2 iNSC‐Exo and NSC‐Exo both Inhibit Neuroinflammation and Improve Axonal Outgrowth In Vitro

**Figure 2:**
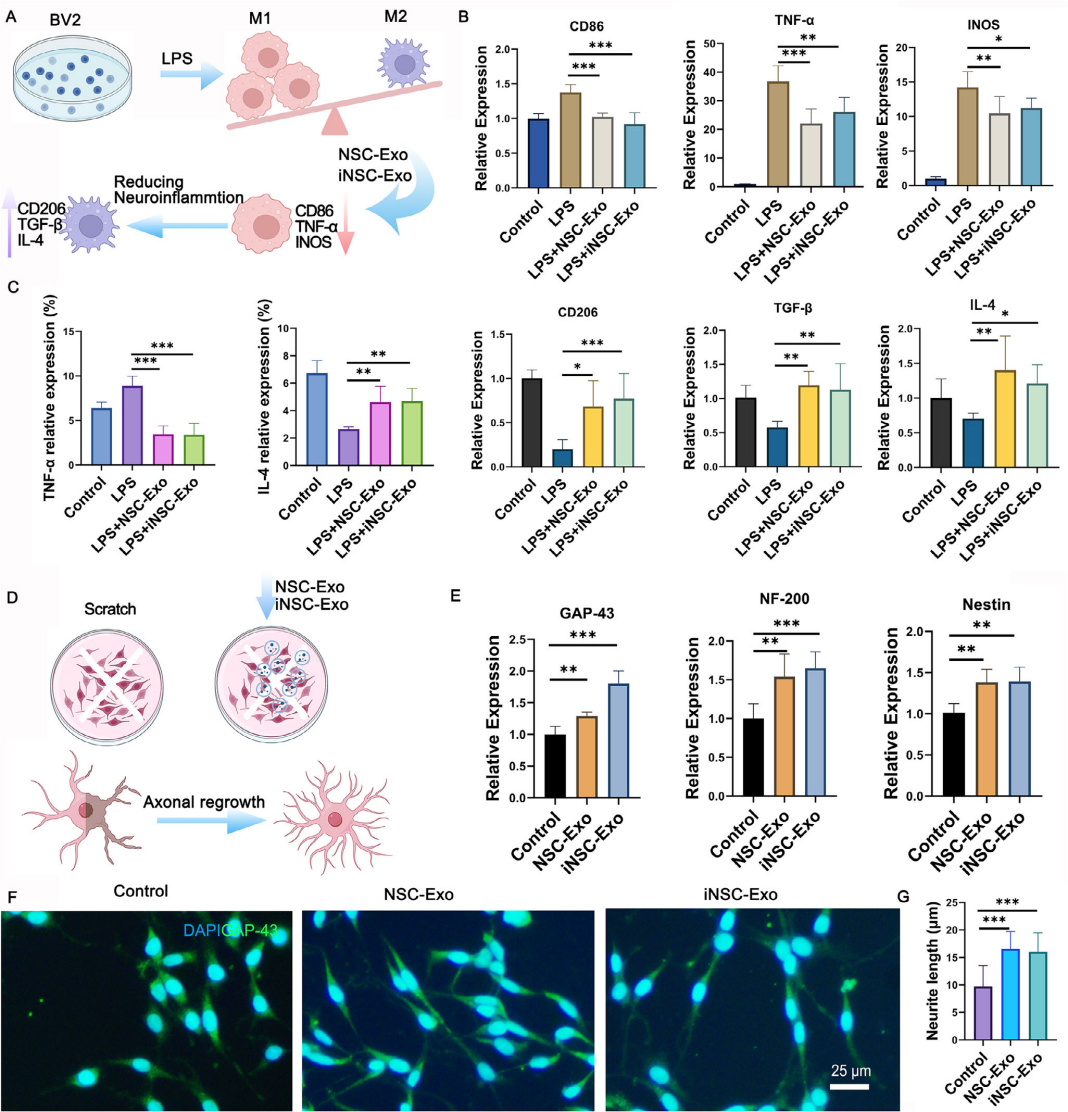
iNSC‐Exo and NSC‐Exo both inhibit neuroinflammation and improve axonal outgrowth in vitro. A) Schematic diagram of the in vitro experiments using BV2 cells. B) The transcript expression of CD86, TNF‐α, iNOS, CD206, TGF‐β, and IL‐4 was determined using Q‐PCR analysis. Data presented as mean ± SD, *n* = 6, *P*‐values are calculated using one‐way ANOVA with Tukey's multiple‐comparisons test, *P < 0.05, **P < 0.01, ***P < 0.001. C) Flow cytometry to detect the relative expression of TNF‐α and IL‐4. Data presented as mean ± SD, *n* = 6, *P*‐values are calculated using one‐way ANOVA with Tukey's multiple‐comparisons test, **P < 0.01, ***P < 0.001. D) Schematic diagram of the in vitro experiments using PC12 cells. E) The transcript expression of GAP‐43, NF‐200, and Nestin using Q‐PCR analysis. Data presented as mean ± SD, *n* = 6, *P*‐values are calculated using one‐way ANOVA with Tukey's multiple‐comparisons test, **P < 0.01, ***P < 0.001. F) Representative immunofluorescence images of GAP‐43 and quantification of the neurite length (*n* = 50) (G). Data presented as mean ± SD, *n *= 50, *P*‐values are calculated using one‐way ANOVA with Tukey's multiple‐comparisons test, ***P < 0.001.

To study the potential impact of exosomes on microglial polarization, lipopolysaccharide (LPS)‐treated BV2 cells were cocultured with NSC‐Exo and iNSC‐Exo (**Figure** **2**A). The mRNA levels of M1 (e.g., CD86, TNF‐*α*, and INOS) and M2 (e.g., CD206, TGF‐β, and IL4) polarization markers were determined using Q‐PCR analysis. LPS treatment upregulated M1 markers and downregulated M2 markers in the BV2 cells. After NSC‐Exo or iNSC‐Exo treatment, LPS‐induced BV2 cells exhibited reduced expression of M1 markers and increased expression of M2 markers (Figure 2B). In addition, both exosome‐treated groups exhibited lower levels of proinflammatory cytokines (e.g., TNF‐*α*) and higher levels of anti‐inflammatory cytokines (e.g., IL4) compared to the LPS‐only group using flow cytometry (Figure 2C; Figure S4, Supporting Information). To model mechanical trauma to the CNS in murine subjects, PC12 cells were subjected to a scratch injury and cocultured with NSC‐Exo and iNSC‐Exo (Figure 2D). Nestin (an intermediate filament protein associated with early stages of development in the central and peripheral nervous systems), neurofilament protein 200 (NF‐200, a neuron‐specific structural protein crucial for axonal transport and maintaining cellular morphology under physiological conditions), and GAP‐43 (commonly associated with axonal processes) were used to assess neurite outgrowth capacity. Q‐PCR results indicated that PC12 cells treated with NSC‐Exo or iNSC‐Exo showed increased Nestin, NF‐200, and GAP‐43 expression, suggesting axonal regeneration (Figure 2E). Similarly, the significantly increased neurite length in the iNSC‐Exo and NSC‐Exo treatment groups revealed that iNSC‐Exo yielded a comparable neuroprotective effect to NSC‐Exo (Figure 2F,G).

### 2.3 Local Delivery of Exosomes to the TBI Defect Can be Achieved after Craniotomy and Using an Injectable Hydrogel

This study fabricated and characterized a novel injectable hydrogel for use as a delivery vehicle for exosomes (Section “Injectable Hydrogel Synthesis and Characterization”). The hydrogel, composed of carboxymethyl chitosan (CMCS), sodium alginate (SA), and supplemented by *D*‐gluconic acid *δ*‐lactone (GDL), exhibited excellent injectability and formed a gel under physiological conditions (Figure 5B; Figure S5A, Supporting Information). Morphological analysis revealed that the hydrogel harbored an interconnected and evenly distributed porous structure, which resulted from the sublimation of H_2_O during lyophilization (Figure S5C, Supporting Information). Fourier‐transform infrared (FTIR) spectroscopy was used for chemical characterization of CMCS, SA, and the lyophilized hydrogel (Figure S5D, Supporting Information). For example, the characteristic peaks at 1626 and 1426 cm^−1^ in CMCS and 1618 and 1413 cm^−1^ in SA corresponded to the asymmetric and symmetric stretching vibrations of the ─COO─ group, respectively. The absorption band ≈3416 cm^−1^ was attributed to the stretching vibration of both ─OH and ─NH groups of CMCS. The bands ≈1035 and 3427 cm^−1^ were attributed to the stretching vibration of the ─OH group of SA. The observed shifts in the peaks at 3443, 1631, and 1454 cm^−1^ indicated that ─NH_2_ in CMCS interacts strongly with GDL as well as ─COOH of SA, suggesting that protonated CMCS interaction with SA increases the mutual binding, which forms a 3D interpenetrating network. Thermodynamically, the TG thermograms of the CMCS, SA, and lyophilized hydrogel samples showed that the first stage ≈60–200 °C, the second stage ≈200–300 °C, and the third stage ≈300–800 °C corresponded to the evaporation of absorbed and bound water, the decomposition of the macromolecule chain cleavage, and further decomposition of the decomposed products, respectively. In addition, the initial thermal decomposition temperatures of SA, CMCS, and hydrogel were 222, 237, and 175 °C, respectively, with their thermal decomposition rates observed in a descending order (Figure S5E,F, Supporting Information). Finally, the gelling behavior of the hydrogel solution was investigated by measuring the storage modulus (G′) and loss modulus (G″) as a function of time. The gelation time of the CMCS/SA (1:1) solution was 547 ± 98 s (Figure S5G, Supporting Information, when incubated at 20 °C), confirming its feasibility for injection.

To analyze the retention and release of exosomes in vitro, the BCA protein assay was used to assess the release behavior of Milk‐Exo from the injectable hydrogel. Most exosomes followed a sustained‐release profile, with ≈91.45 ± 16.27% being released into PBS over a period of 28 days (Figure S5H, Supporting Information). This sustained release of exosomes from hydrogels was attributed to two primary mechanisms, that is, diffusion facilitated by hydrogel swelling and gradual polymer erosion. The in vivo retention of exosomes was analyzed by acquiring fluorescence images of murine subjects using an IVIS Spectrum system (Figure S6, Supporting Information). By day 14, the injection group exhibited significantly less residual Milk‐Exo compared to the implantation group. Furthermore, from day 21 onward, Milk‐Exo were nearly eliminated in the injection group but persisted in the implantation group. Collectively, hydrogel‐mediated protection and controlled release synergistically extended the retention of exosomes in vivo and enhanced long‐term therapeutic efficacy. Therefore, the hydrogel implantation group demonstrated a sustained and progressively increasing DiR fluorescence signal from day 0 to day 14. These findings suggested that the hydrogel effectively prevented the clearance of exosomes and prolonged their retention time in vivo. Finally, the in vivo degradation analysis revealed that the hydrogel was degraded over 4 weeks after being injected in situ into the surgical area of C57BL/6 mice (Figure S7A,B, Supporting Information). Histological micrographs of the brain tissue in contact with the implanted hydrogel at different time intervals are shown in Figure S7C (Supporting Information). No signs of an inflammatory reaction were observed in the surrounding tissues, indicating excellent biocompatibility and biodegradability of the hydrogel.

### 2.4 Local Delivery of iNSC‐Exo Can Protect the Blood–Brain Barrier, Reduce Brain Edema, and Promote Functional Recovery after TBI in Mice

**Figure 3:**
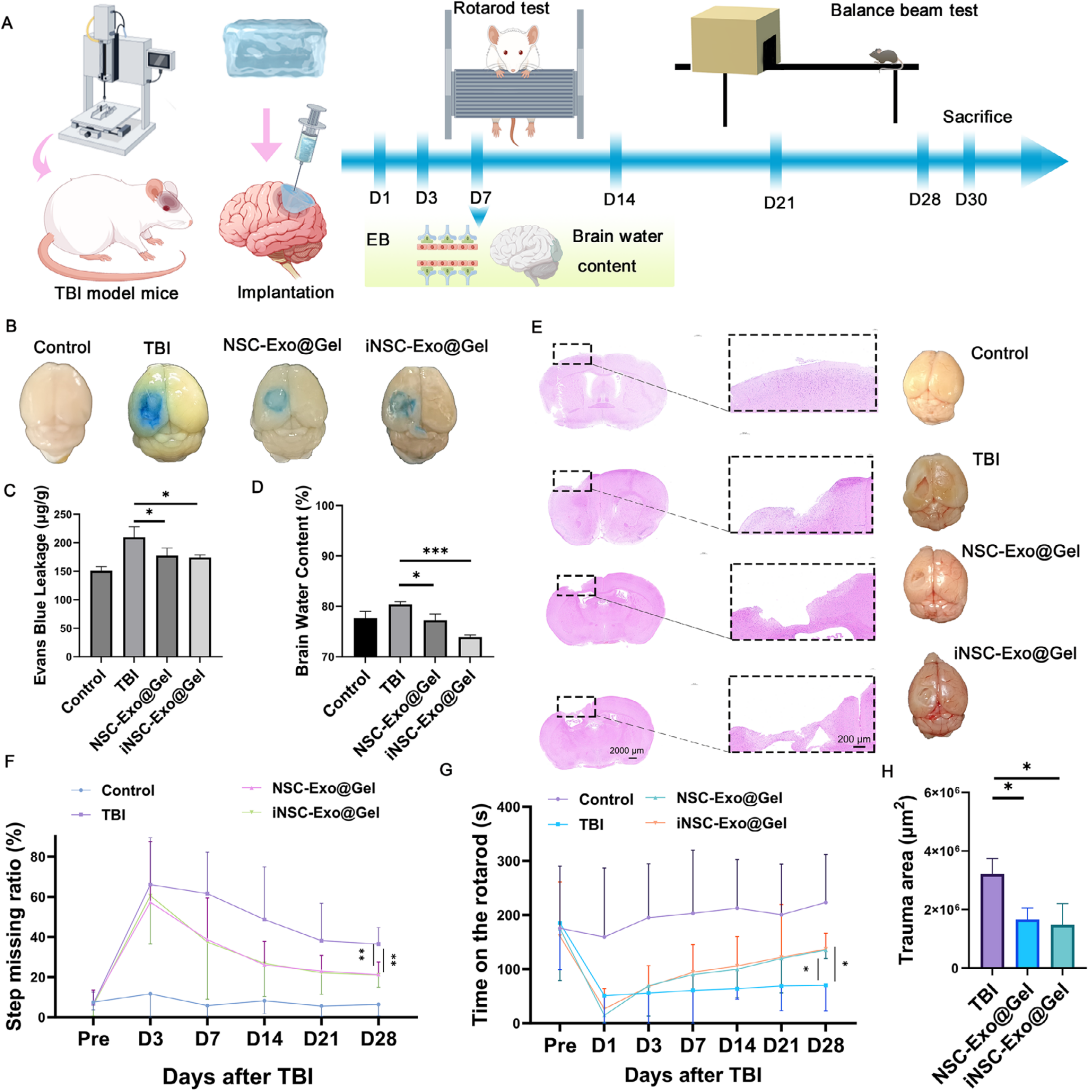
Local delivery of exosomes to the TBI defect can be achieved after craniotomy and using an injectable hydrogel. A) Schematic illustration of modelling procedures, treatment administration, and behavioral tests. B) Representative pictures of EB extravasation in the brains of various groups at day 7 after TBI. C) Quantitative analysis of EB leakage in each group. Data presented as mean ± SD, *n* = 3, *P‐*values are calculated using one‐way ANOVA with Tukey's multiple‐comparisons test, *P < 0.05. D) Brain water content change before and after exosomal treatment. Data presented as mean ± SD, *n* = 3, *P*‐values are calculated using one‐way ANOVA with Tukey's multiple‐comparisons test, *P < 0.05, ***P < 0.001. E) H&E images and representative pictures showing the brain defects at 28 days post‐surgery. F) The step missing ratio on days 3, 7, 14, 21, and 28. Data presented as mean ± SD, *n* = 6–9, *P*‐values are calculated using two‐way repeated‐measures ANOVA with Tukey's multiple‐comparisons test, **P < 0.01. G) Rotarod performances of various groups from day 1 to day 28. Data presented as mean ± SD, *n* = 6–9, *P*‐values are calculated using two‐way repeated‐measures ANOVA with Tukey's multiple‐comparisons test, *P < 0.05. H) The trauma area was quantified. Data presented as mean ± SD, *n* = 3, *P*‐values are calculated using one‐way ANOVA with Tukey's multiple‐comparisons test, *P < 0.05.

To comprehensively validate the therapeutic effect of locally delivered iNSC‐Exo using an injectable hydrogel (iNSC‐Exo@Gel), a series of neurological experiments was performed (**Figure** **3**A). TBI often leads to significant disruption of the blood‐brain barrier (BBB), resulting in brain edema and other severe complications. First, to assess BBB permeability post‐TBI, an Evans Blue (EB) extravasation test was conducted. On day 7 post‐TBI, either NSC‐Exo@Gel or iNSC‐Exo@Gel treatment could reduce BBB permeability in the injured regions (Figure 3B,C), indicating that both hydrogel‐loaded NSC‐Exo and iNSC‐Exo could preserve BBB integrity with equivalent efficacy. Similarly, treatments with NSC‐Exo@Gel or iNSC‐Exo@Gel significantly reduced brain water content, indicating their potential anti‐edema effects (Figure 3D). Finally, H&E staining revealed significant attenuation of TBI‐induced tissue damage in both exosome treatment groups (Figure 3E,H).

Next, behavioral assessments were conducted to confirm the functional recovery of TBI mice treated with NSC‐Exo@Gel or iNSC‐Exo@Gel. The balance beam test and rotarod test were used to evaluate motor balance and coordination. The results indicated that both iNSC‐Exo@Gel and NSC‐Exo@Gel treatments could progressively reduce the missed step ratio across all measured timepoints (days 7, 14, 21, and 28, Figure 3F), suggesting enhanced motor recovery. Similarly, both exosomal treatments demonstrated enhanced rotarod performance, with a 1.5‐fold increase compared to the TBI group (Figure 3G).

Finally, histological evidence was obtained to further characterize the therapeutic effects. The neuroinflammatory response to TBI plays a pivotal role in the prognosis of secondary injury. While TBI increased the levels of ionized calcium‐binding adapter molecule 1 (Iba‐1) and glial fibrillary acidic protein (GFAP), which are specifically expressed by microglia and astrocytes, respectively, either NSC‐Exo@Gel or iNSC‐Exo@Gel treatment could suppress neuroinflammation by reducing the expression of these two markers (Figure S8, Supporting Information). Meanwhile, mice treated with locally delivered exosomes showed a significant upregulation of GAP‐43 and DCX, which are associated with axonal processes and neuronal migration, respectively, suggesting that both NSC‐Exo@Gel and iNSC‐Exo@Gel treatments comparably facilitated nerve regeneration after TBI (Figure S8, Supporting Information).

Collectively, the behavioral tests and histological studies demonstrated excellent therapeutic potential of locally delivered iNSC‐Exo using an injectable hydrogel during the treatment of TBI, with comparable efficacy to NSC‐Exo@Gel. These results were further supported by a systemic biosafety assessment. H&E staining showed no evidence of significant organ damage with the application of NSC‐Exo@Gel or iNSC‐Exo@Gel (Figure S9A, Supporting Information). Similarly, liver function indicators (e.g., aspartate aminotransferase [AST] and alanine aminotransferase [ALT]) and renal function markers (e.g., creatinine [CREA] and urea [UREA]) remained unchanged, confirming that neither NSC‐Exo@Gel nor iNSC‐Exo@Gel adversely affected liver or kidney functions (Figure S9B, Supporting Information).

### 2.5 RVG‐Modified Strategy Enhances iNSC‐Exo Enrichment via Systemic Administration in TBI Mouse Brains In Vivo

**Figure 4:**
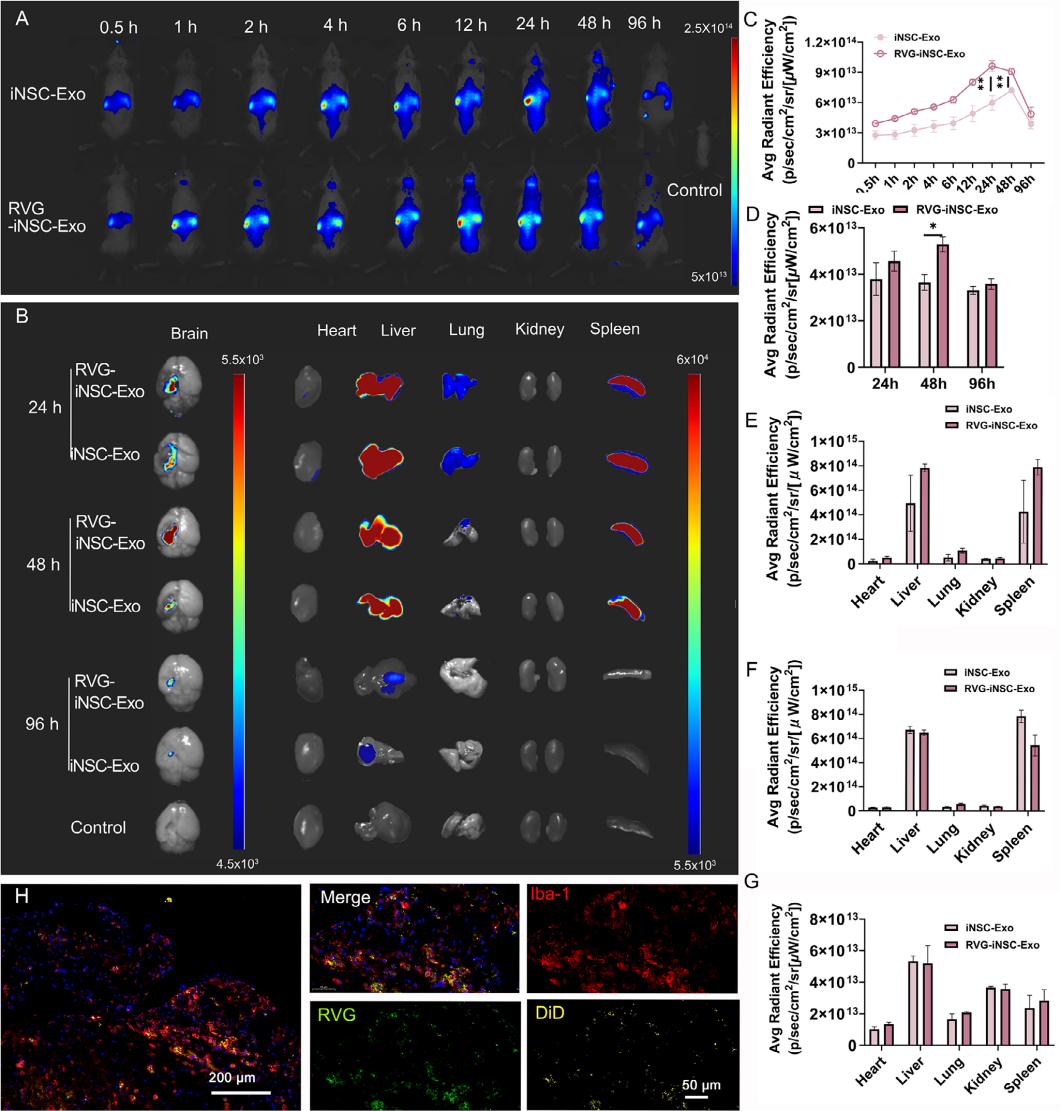
Modifying exosomes with RVG can enhance the enrichment of iNSC‐Exo in the brains of mice with TBI after systemic administration. A) DiD fluorescence images of TBI mice 0.5, 1, 2, 4, 6, 12, 24, 48, and 96 h after intravenous injection of exosomes. B) DiD fluorescence images of brain, heart, liver, lungs, kidneys, and spleen ex vivo 24, 48, and 96 h after intravenous injection of exosomes. C) DiD fluorescence intensity of the brain, based on Figure 4A. Data presented as mean ± SD, *n* = 5, *P*‐values are calculated using two‐way repeated‐measures ANOVA with Tukey's multiple‐comparisons test, **P < 0.01. D) DiD fluorescence intensity of the brain, based on Figure 4B. Data presented as mean ± SD, *n *= 3, *P*‐values are calculated using two‐way ANOVA with Tukey's multiple‐comparisons test, *P < 0.05. DiD fluorescence intensity of major organs 24 h (E), 48 h (F), and 96 h (G) after intravenous injection base on Figure 4B. H) Brain sections of TBI mice marked with microglia‐labelling Iba‐1 (red) with nuclei stained with DAPI (blue). RVG‐iNSC‐Exo was labeled with FITC (green) and DiD (yellow).

As previously mentioned (Section 1), only 10% of TBI patients presenting with moderate‐to‐severe disease require surgical intervention; the majority are managed conservatively or medically.^[^ ^17^ ^]^ Therefore, systemic administration of drugs or products via intravenous injection constitutes a primary treatment modality. Although exosomes can cross the BBB mainly due to their nanoscale size, little accumulation is detected in the brain after systemic administration, with most of the administered exosomes found in the spleen and liver.^[^ ^21^ ^]^ To overcome this obstacle and improve exosome accumulation in the CNS, brain‐targeting exosomes were generated by modifying iNSC‐Exos with RVG peptide, which interacts specifically with the acetylcholine receptor to facilitate viral entry into neuronal cells in the CNS.^[^ ^22^ ^]^ In our study, RVG‐modified induced neural stem cell‐derived exosomes (RVG‐iNSC‐Exo) were obtained with a preparative yield of ≈50% using the bio‐orthogonal click chemistry technique. Following tail vein injection, whole‐body, live, fluorescence imaging at different time points revealed brain fluorescence signals based on DiD labeling, with the RVG‐iNSC‐Exo group demonstrating a significantly stronger signal in the brain compared to unmodified iNSC‐Exo (**Figure** **4**A). Furthermore, a quantitative analysis was consistent with these findings (Figure 4C), suggesting superior targeting ability of RVG‐iNSC‐Exo toward the brain in vivo. Similarly, fluorescence imaging and quantification of the excised brains further indicated higher accumulation of RVG‐iNSC‐Exo compared to unmodified exosomes at 24 and 48 h post‐injection (Figure 4B,D). Besides, these exosomes mainly accumulated in the liver and spleen, with a small amount in the lungs, both qualitatively and quantitatively (Figure 4E–G). Finally, the co‐localization of DiD and FITC signals with the immunoreactivities of Iba‐1 indicated the uptake of RVG‐iNSC‐Exo by microglial cells in the mouse brains (Figure 4H).

### 2.6 Systemic Administration of RVG‐Modified iNSC‐Exo Can Improve Motor Functions, Increase Cognitive Capacity, and Enhance Cognitive Recovery after TBI in Mice

**Figure 5:**
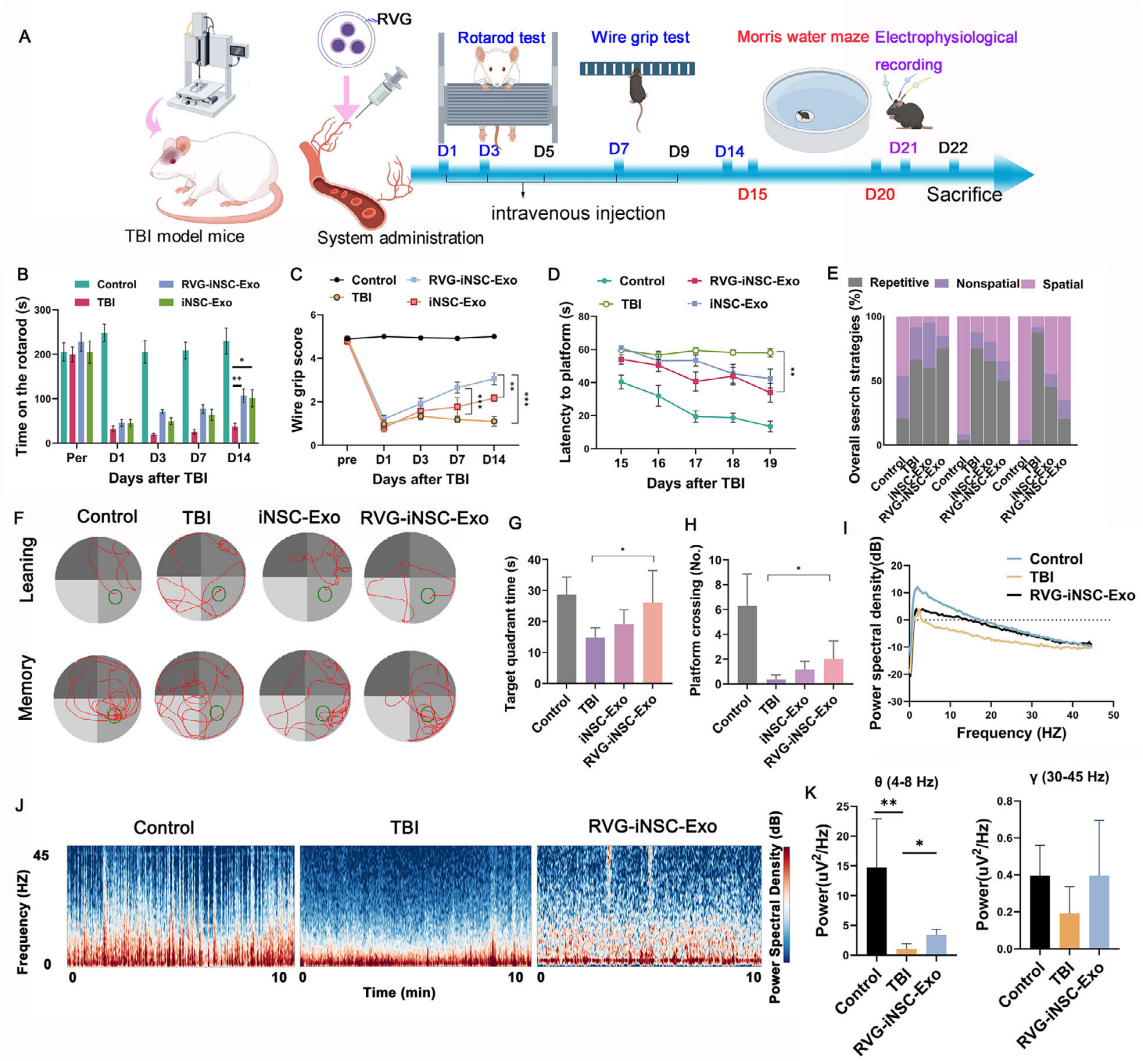
Systemic administration of iNSC‐Exo can be enhanced using RVG modification of the exosomes, thereby improving motor functions, increasing cognitive capacity, and enhancing cognitive recovery after TBI in mice. A) Schematic timeline of the experimental procedures. B) Rotarod performances of mice in various treatment groups. Data presented as mean ± SD, *n =* 6–9, *P*‐values are calculated using two‐way repeated‐measures ANOVA with Tukey's multiple‐comparisons test, *P < 0.05, **P < 0.01. C) Wire grip score of mice in various treatment groups. Data presented as mean ± SD, *n* = 6–9, *P*‐values are calculated using two‐way repeated‐measures ANOVA with Tukey's multiple‐comparisons test, **P < 0.01, ***P < 0.001. D) Escape latencies in the learning phase. Data presented as mean ± SD, *n* = 6–9, *P*‐values are calculated using two‐way repeated‐measures ANOVA with Tukey's multiple‐comparisons test, **P < 0.01. E) Distribution of search strategies. F) Computer printouts of the swimming trajectories of mice in the Morris water maze test. G) Time spent in the target quadrant and H) number of platform crossings in the memory phase. Data presented as mean ± SD, *n* = 6–9, *P*‐values are calculated using one‐way ANOVA with Tukey's multiple‐comparisons test, *P< 0.05. I) Power spectra of LFP in the hippocampus. J) Time frequency diagrams of hippocampus activity. K) Quantification of average theta and gamma band power in the hippocampus. Data presented as mean ± SD, *n* = 3, *P*‐values are calculated using one‐way ANOVA with Tukey's multiple‐comparisons test, *P < 0.05, **P < 0.01.

To investigate the therapeutic potential of RVG‐iNSC‐Exo in TBI mice, the treatment protocol and evaluations were conducted based on the timeline presented in **Figure** **5**A. First, the RVG‐iNSC‐Exo‐treated TBI mice exhibited superior motor recovery in the rotating rod test compared to other groups (Figure 5B). The wire grip test, which employs a scoring system ranging from 0 to 5 as detailed in Table S2 (Supporting Information), further corroborated these findings, with RVG‐iNSC‐Exo‐treated mice exhibiting enhanced muscle strength and reduced motor asymmetry (Figure 5C).

Next, the Morris water maze test was conducted to evaluate the cognitive functions of learning and memory abilities of mice after TBI. As shown in Figure 5D, the escape latency progressively decreased over the five‐day training period. Compared to the untreated TBI mice, the iNSC‐Exo and RVG‐iNSC‐Exo‐treated TBI mice required a shorter time to find the hidden platform. Interestingly, the RVG‐iNSC‐Exo group showed significantly improved performance in escape latency over the five training days relative to the iNSC‐Exo group. These findings suggested that RVG‐iNSC‐Exo can effectively enhance cognitive recovery following TBI. Furthermore, the selection of spatial search strategies serves as a crucial indicator of the development of an allocentric map to the escape location during spatial learning. These strategies can be classified as non‐spatial, spatial, and repetitive searching strategies.^[^ ^23^ ^]^ After TBI, mice demonstrated a reduced ability to learn these strategies, which was reflected by a higher percentage of repetitive searching strategies compared to control mice. The RVG‐iNSC‐Exo group experienced a larger ratio of spatial strategy than the TBI groups, suggesting that RVG‐iNSC‐Exo‐treated TBI mice exhibited higher cognitive capacity (Figure 5E). On day 6 of the Morris water maze test, a probe trial was performed without the platform to evaluate memory consolidation. Besides, the results showed that mice in the RVG‐iNSC‐Exo group could remember the position of the platform, as evidenced by their swimming paths, which were more direct compared to the chaotic paths of the untreated TBI group. The RVG‐iNSC‐Exo group spent more time in the target quadrant and increased the frequency of platform crossings compared to the TBI group (Figure 5F–H). The local field potential (LFP) represents the combined electrical activity within neural tissue. TBI can lead to significant disruptions in neuronal structures such as the cell body and axon, which may impede the normal transmission of electrical signals and alter the internal environment of neurons. Consequently, these changes can affect the frequency, amplitude, and other electrical properties of the LFP. LFP can be classified into different bands according to the frequency, including delta (*δ*, 0.5–4 Hz), theta (*θ*, 4–8 Hz), alpha (*α*, 8–13 Hz), beta (*β*, 13–30 Hz), and gamma (*γ*, 30–45 Hz), with different frequency bands reflecting different states of the brain (Table S3, Supporting Information).^[^ ^24^, ^25^ ^]^ In the TBI group, there was a significant reduction in oscillation power across the 0–45 Hz range compared to the control group. Notably, treatment with RVG‐iNSC‐Exo partially restored the diminished oscillation power within this frequency range (Figure 5I,J). The hippocampus is integral to learning and memory processes, and *θ* and *γ* oscillations have been associated with cognitive functions related to these processes.^[^ ^26^ ^]^ Our study results suggested that systemic administration of RVG‐iNSC‐Exo could rescue TBI‐induced disruption of *θ* and *γ* activities during a cognitive task (Figure 5K; Figure S10, Supporting Information).

Finally, a photograph examination of brain tissue revealed substantial structural defects in the TBI groups (Figure S11A, Supporting Information). However, the RVG‐iNSC‐Exo group demonstrated markedly preserved tissue architecture. To further validate these findings, we performed quantitative analysis of the lesion area using H&E‐stained sections (Figure S12, Supporting Information). The results consistently demonstrated that the RVG‐iNSC‐Exo group and the iNSC‐Exo group exhibited significantly reduced tissue damage compared to the TBI group, corroborating our previous observations. Further H&E staining images and serum biomarker testing confirmed the safety profile of RVG‐iNSC‐Exo, with no evidence of toxicity to major organs (Figure S11B,C, Supporting Information).

### 2.7 Single‐Cell Sequencing Reveals that Microglia are the Predominant Type of Cells Affected by iNSC‐Exo Treatment in the Brains of Mice with TBI

**Figure 6:**
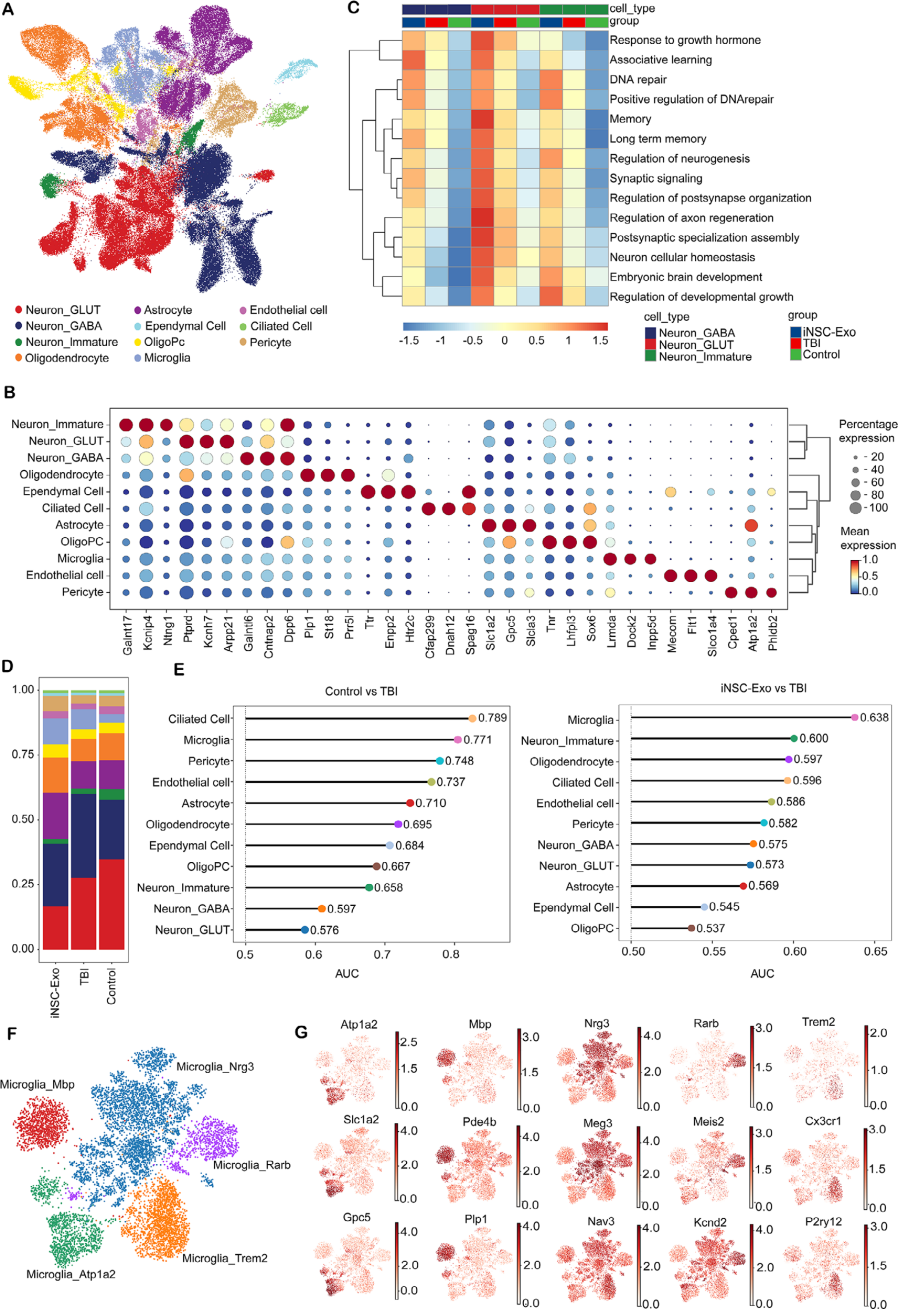
Single‐nucleus cell analysis demonstrated that microglia are the predominant type of cells affected by iNSC‐Exo treatment in the brains of mice with TBI. A) The visualization of eleven distinct cell classes was achieved using Uniform manifold approximation and projection (UMAP). Each colored sphere corresponds to a specific cell class, with individual dots representing single cells (*n* = 9 for cortex and hippocampus, 3 for control, 3 for TBI, and 3 for iNSC‐Exo). B) Dot plot illustrating the gene expression signature for each individual cell type. C) GSVA reveals differential pathway activity across three neuron subpopulations among the three groups. D) Bar chart illustrating the distribution of 11 cell types in three groups. E) Lollipop plot utilizing Augur demonstrates cells exhibiting the most significant variability, as determined by AUC values, in association with TBI when compared to the control group and iNSC‐Exo group. F) The UMAP analysis displays all cells with different colors, indicating 5 microglial subpopulations. G) UMAP plots illustrating the expression profiles of cell type‐specific marker genes for the respective microglial subpopulations.

To elucidate the fundamental mechanisms underlying the therapeutic effects of iNSC‐Exo following TBI, single‐cell nuclei RNA sequencing (snRNA‐seq) was performed on cortical and hippocampal tissues from mice assigned to the control, TBI, and iNSC‐Exo groups on day 30 post‐injury. A total of 113 802 cells were analyzed, including 37 767 from the control group, 42 240 from the TBI group, and 33 795 from the iNSC‐Exo group. The analysis identified 11 distinct cell types, each characterized by unique expression profiles, for example, microglia, immature neurons, oligodendrocytes, ciliated cells, pericytes, GABAergic neurons, glutamatergic neurons, astrocytes, ependymal cells, endothelial cells, and oligodendrocyte precursor cells (**Figure** **6**A). Furthermore, differential enrichment of known marker gene transcripts was observed across these cell types (Figure 6B).

Single‐cell transcriptomes were organized into clusters, and cell types were identified based on known markers and established evidence.^[^ ^27^, ^28^ ^]^ Gene set variation analysis (GSVA) revealed that relative to the TBI group, the iNSC‐Exo treatment group exhibited a markedly enhanced enrichment of pathways associated with neural regeneration, embryonic brain development, learning, and memory across three neuronal subtypes. In addition, these pathways demonstrated even greater prominence in the iNSC‐Exo treatment group when compared to the TBI group (Figure 6C).

Utilizing cellular proportion analysis, an increased proportion of microglia, oligodendrocytes, and astrocytes was identified in the brains of mice subjected to TBI and subsequent treatment with iNSC‐Exo, with the treatment group exhibiting a notably higher proportion (Figure 6D; Figure S13A,B, Supporting Information). To ascertain the cellular populations most significantly impacted at the site of brain injury post‐iNSC‐Exo treatment, Augur analysis was conducted, revealing that microglia were the most prominent cell type in the iNSC‐Exo treatment group relative to the TBI group, while showing significant alterations in the TBI group compared to the treatment group (Figure 6E; Figure S13C, Supporting Information). Accordingly, our subsequent investigations focused on microglia. Subpopulation analysis revealed the segregation of microglia into five distinct subpopulations, as visualized via a UMAP plot (Figure 6F). These subpopulations were designated based on their characteristic genes, that is, Microglia_Nrg3, Microglia_Mbp, Microglia_Rarb, Microglia_Atp1a2, and Microglia_Trem2, with the specific genes of each subpopulation depicted in the UMAP plots (Figure 6G).

### 2.8 Microglia_Nrg3 and Microglia_Rarb Subpopulations with Reduced Differentiation State Mediate Neural Repair Functions via iNSC‐Exo Treatment

**Figure 7:**
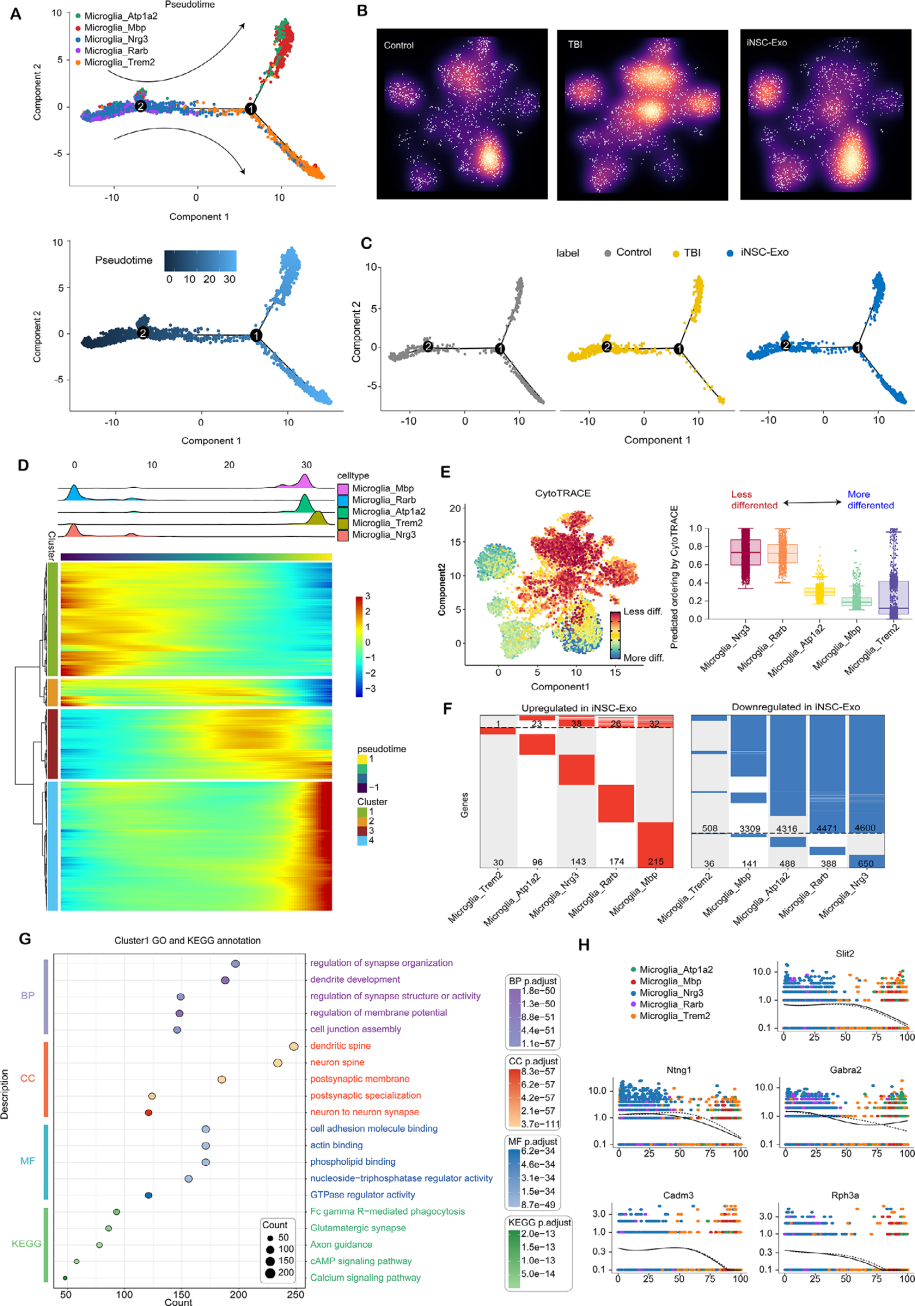
Microglia_Nrg3 and Microglia_Rarb subpopulations that exhibit a reduced state of differentiation could be responsible for neural repair functions by iNSC‐Exo treatment. A) The prospective developmental pathways of five microglial subsets were inferred using Monocle. Each dot represents an individual cell, with colors indicating the specific subsets (top) and pseudotime (bottom). B) Galaxy plots illustrate the cell density within the UMAP space for microglial subpopulations across three groups: 1083 control (left), TBI (middle), and iNSC‐Exo (right). Cooler colors represent regions of lower density, whereas warmer colors signify regions of higher density. C) The trajectory of microglia subpopulations, analyzed using Monocle, is categorized into three groups. D) Ridge plot illustrates the distinct gene expression patterns of each microglial subpopulation throughout the pseudotime trajectory, while the accompanying heatmap categorizes these gene expression patterns into four distinct clusters. E) The differentiation status of each microglial subpopulation was assessed using CytoTRACE analysis. F) The differential upregulation and downregulation of genes in microglial subpopulations were observed in the iNSC‐Exo group in comparison to the TBI group. G) The GO and KEGG bubble plot display the enrichment results of cluster 1 genes from the heatmap in (D). H) Spline plots illustrate the expression kinetics of five exemplar genes associated with synaptic or axon formation across pseudotime.

To further elucidate the distinctions among these microglial subpopulations, a pseudotime analysis was conducted, which unveiled a unique differentiation trajectory among the five microglial subtypes. Notably, Microglia_Nrg3 and Microglia_Rarb were predominantly situated at the initial developmental stage on the left side of the trajectory, whereas the remaining three subpopulations were primarily concentrated in the intermediate and terminal phases of the developmental trajectory (**Figure** **7**A; Figure S14, Supporting Information). Analysis of the density distribution and developmental trajectory characteristics of the microglia subpopulations revealed that the iNSC‐Exo treatment group and the control group demonstrated analogous cell density patterns and developmental trajectories. Specifically, both groups exhibited elevated cell density at the Microglia_Trem2 position, with a greater abundance of cells in the lower‐right branch of the trajectory. Conversely, the TBI group showed increased cell density at the Microglia_Nrg3 and Microglia_Rarb positions, with a reduced number of cells in the lower‐right branch (Figure 7B,C; Figure S13D, Supporting Information). A comprehensive analysis of gene expression patterns along the pseudotime trajectory revealed that Microglia_Nrg3 and Microglia_Rarb exhibited elevated gene expression levels at the initial phase of pseudotime, as depicted in the Ridge plot (Figure 7D).

Furthermore, heatmap analysis identified four distinct clusters of gene expression based on temporal variations (Figure 7D). To substantiate the less differentiated state of Microglia_Nrg3 and Microglia_Rarb, a CytoTRACE analysis was performed, which yielded higher scores for these subpopulations (Figure 7E). Comparative analysis of the upregulated and downregulated genes in these microglial subpopulations between the iNSC‐Exo and TBI groups revealed that Microglia_Nrg3 and Microglia_Rarb exhibited the most pronounced differences in gene expression among the five subpopulations, suggesting the potential functional importance of the identified DEGs (Figure 7F).

Building upon these findings, gene ontology (GO) and Kyoto Encyclopedia of Genes and Genomes (KEGG) enrichment analyses were conducted on the genes within cluster 1, as depicted in Figure 7G. These genes exhibited elevated expression levels in the Microglia_Nrg3 and Microglia_Rarb subpopulations. The analyses revealed a significant enrichment of genes involved in synaptic and axonal signaling pathways. Besides, the pseudotime distribution analysis of several canonical synaptic‐related genes indicated their pronounced expression at the early stages of pseudotime (Figure 7H). This suggested that these two microglia subpopulations might have functional roles associated with synaptic and axonal processes.

### 2.9 iNSC‐Exo Treatment Can Improve Synaptic Connectivity between Microglia and Neurons via Activation of NRXN Signaling Pathways

**Figure 8:**
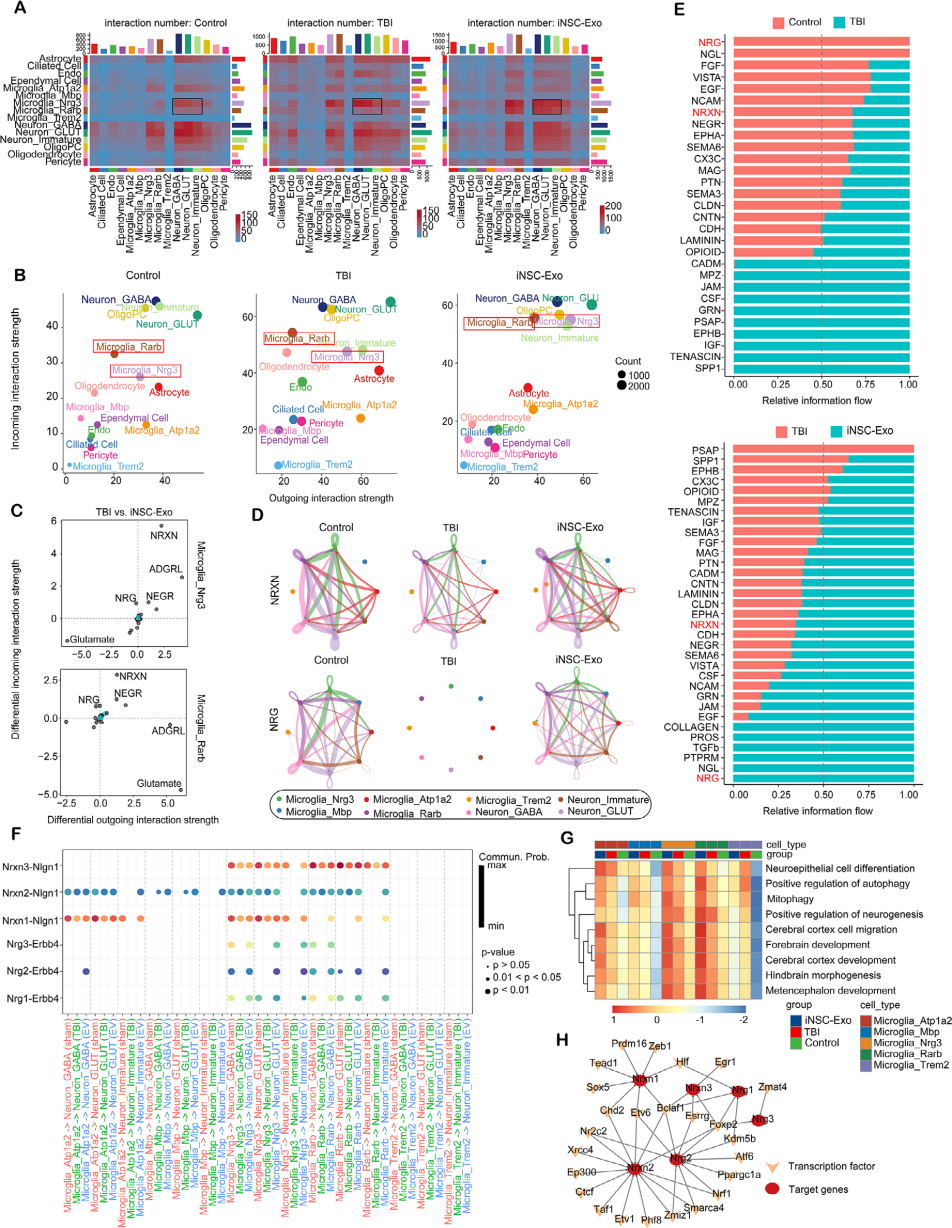
iNSC‐Exo treatment possesses the potential to improve synaptic connectivity between microglia and neurons through the activation of NRXN signaling pathways. A) The heatmap shows the difference in the number of cell communications of each cell population. B) Variations in signal reception and transmission among control, TBI, and iNSC‐Exo groups. C) Variations in signaling changes of Microglia_Nrg3 and Microglia_Rarb among TBI and iNSC‐Exo groups. D) The NRXH and NRG signaling networks within the control, TBI, and iNSC‐Exo groups. E) Stacked charts illustrate the distinctions in intercellular signaling pathways among the control, TBI, and iNSC‐Exo groups. F) Dot plot represents the disparity in signaling molecules from Microglia_Nrg3 to neurons among the control, TBI, and iNSC‐Exo groups. G) GSVA reveals differential pathway activity across three neuron subpopulations among three groups. H) The gene regulatory networks of NRXN and NRG were deduced using the SCENIC method.

Subsequently, a CellChat analysis was conducted to examine the variations in cell–cell communication among the control, TBI, and iNSC‐Exo groups. Our observations indicated an increase in the number of cellular interactions in both the TBI and iNSC‐Exo groups, with a more substantial enhancement observed in the iNSC‐Exo group (Figure S13E,F, Supporting Information). Further analysis identified Microglia_Nrg3 and Microglia_Rarb as important signal senders to neurons, which functioned as signal receivers, across all three groups. Notably, these interactions were more pronounced in the TBI and iNSC‐Exo groups (**Figure** **8**A). This finding was further substantiated by the weight diagram illustrating the sending and receiving strength of signals, which indicated that Microglia_Nrg3 and Microglia_Rarb exhibited enhanced signal transmission and reception with neurons in the iNSC‐Exo group (Figure 8B).

To further substantiate the interactions between Microglia_Nrg3/Microglia_Rarb and neurons, we conducted immunofluorescence staining on brain sections from the three experimental groups and quantified the distances between Nrg3+/Rarb+ non‐neuronal cells and NeuN+ neurons. Quantitative analysis revealed that, in comparison to the Control group, both the TBI and iNSC‐Exo groups demonstrated a higher proportion of Nrg3+/Rarb+ non‐neuronal cells located within 0–20 and 0–40 µm of NeuN+ neurons. Notably, the iNSC‐Exo group exhibited an even greater proportion of cells within these interaction distances (Figure S15, Supporting Information), further corroborating the cell communication patterns previously identified.

To further investigate the potential signals of significant influence, we analyzed the signal proportion for TBI and iNSC‐Exo groups. Notably, the NRXN and NRG signals were increased in the iNSC‐Exo group relative to the TBI group (Figure 8C). A more comprehensive examination of the NRXN and NRG signaling in the interaction between microglia and neurons also elucidated this phenomenon (Figure 8D). Subsequently, a comparative analysis of signal flow differences was conducted among the three groups, focusing on the interaction of microglia with neurons. This analysis further corroborated that the NRXN and NRG signaling pathways exhibited significant variations between the TBI and iNSC‐Exo groups (Figure 8E).

Accordingly, the strength of intercellular ligand–receptor interactions between each microglia subpopulation and neurons was examined using a diagrammatic representation. Our investigation revealed that Nrxn1‐3 originating from Microglia_Nrg3 and Microglia_Rarb interacted with various synaptic proteins in neurons, including Nlgn1. Moreover, these receptor–ligand interactions were attenuated following TBI and subsequently reinforced after iNSC‐Exo treatment. Besides, the receptor–ligand interactions between Nrg1‐3 and Erbb44 demonstrated comparable variations in interaction strength across the three groups, similar to those observed with Nrxn1‐3‐Nlgn1 (Figure 8F). GSVA further corroborated that the Microglia_Nrg3 and Microglia_Rarb subpopulations demonstrated enhanced regulation of functions related to neural regeneration, development, and autophagy after iNSC‐Exo treatment (Figure 8G). An analysis of transcription factor regulation identified two transcription factors, including Bclaf1 and Foxp2, that may regulate the expression of both NRXN and NRG. These TFs shared higher regulon activity within the Microglia_Nrg3 and Microglia_Rarb subpopulations in the iNSC‐Exo group (Figure 8H; Figure S16, Supporting Information). This discovery potentially elucidated the positive influence of these transcription factors in modulating NRXN and NRG signaling after iNSC‐Exo treatment.

## 3 Discussion

Globally, TBI accounts for millions of fatalities annually and remains a predominant cause of mortality and disability, especially among young individuals.^[^ ^29^ ^]^ Given the irreversibility of the primary injury, the prevention of secondary injury is of paramount importance in clinical practice. Treatment options vary significantly depending on the severity of TBI, and primarily include surgery (e.g., craniotomy in the acute phase for the severe cases) and medicine (e.g., selective serotonin reuptake inhibitors in the post‐acute phase for the mild ones).^[^ ^30^ ^]^ There is currently no universal therapeutic agent applicable across both surgical and medical contexts of TBI management. To the best of our knowledge, this study represents the first application of iNSC‐Exo in the treatment of TBI. Our comprehensive work aimed to simulate the clinical practice using a murine model and mainly focused on three aspects. First, we confirmed that the iNSC‐Exo could promote the recovery of TBI and revealed their therapeutic comparability to NSC‐Exo. Besides, we established two clinically inspiring administration modalities of iNSC‐Exo, that is, local delivery through craniotomy and systemic delivery through venous injection and enhanced their efficacy via injectable hydrogel and RVG targeting, respectively. Finally, the underlying mechanism of iNSC‐Exo therapy was explored using single‐cell sequencing.

Treatment using stem cell‐derived exosomes is now considered a safe, potent, and versatile alternative to conventional stem cell therapy.^[^ ^7^ ^]^ Stem cell‐derived exosomes inherit therapeutic effects from their parental cells, such as tissue regeneration, anti‐inflammation, and immunomodulation,^[^ ^31^ ^]^ without the disadvantages associated with their cellular counterparts.^[^ ^32^ ^]^ Among these, MSC‐Exo and NSC‐Exo have demonstrated tremendous potential in treating various neurological and neurosurgical diseases. Our recent development of mouse fibroblast‐derived iNSCs through somatic reprogramming has provided a high‐yield source of clinical‐grade exosomes,^[^ ^33^ ^]^ thereby creating new opportunities for exosome‐based therapies using NSC‐like cells. Notably, iNSC‐Exo exhibited therapeutic properties comparable to NSC‐Exo, including enhanced cognitive function, attenuated neuroinflammation, and promotion of neuro‐regeneration.^[^ ^34^, ^35^, ^36^ ^]^ These effects have been validated in Alzheimer's disease^[^ ^9^ ^]^ and ischemic stroke.^[^ ^10^ ^]^ While there have been other projects using exosomes for TBI treatment,^[^ ^11^, ^12^, ^14^, ^15^, ^16^, ^37^ ^]^ most have used MSC‐derived exosomes in various animal models. Although MSC‐Exo and NSC‐Exo both yield ameliorative effects on TBI management, these exosomes are widely thought to offer therapeutic benefits. For instance, MSC‐Exos may be more effective for anti‐neuroinflammation, while NSC‐Exos might excel in neuro‐regeneration.^[^ ^38^ ^]^ Therefore, a direct comparison between iNSC‐Exo and other types of SC‐Exo would be of more significant interest for the treatment of TBI.

Local delivery of iNSC‐Exo to the TBI defect was achieved via a craniotomy as an intraoperative procedure in this proof‐of‐concept subproject. Unlike systemic administration, where exosomes are often quickly cleared from blood circulation and accumulate in the liver, spleen, and lungs,^[^ ^39^ ^]^ local delivery of exosomes can improve organ‐specific enrichment of exosomes.^[^ ^40^, ^41^ ^]^ However, even locally delivered exosomes can be easily degraded and become inactive, especially within harsh microenvironments like those found in TBI. Therefore, biomaterials (e.g., hydrogels) have been used in recent years to protect, support, and enhance the effects of locally delivered exosomes, thereby maximizing their therapeutic potential.^[^ ^18^ ^]^ In the present study, we developed a novel injectable hydrogel as a peri‐operative treatment platform aimed at reducing inflammation, facilitating nerve regeneration, and repairing brain tissue structure. This hydrogel offers the following advantages: 1) structurally, it exhibits features similar to the brain tissue extracellular matrix, featuring ultrahigh water content and soft mechanical properties, thereby creating an optimal microenvironment for TBI; 2) pharmacologically, the hydrogel serves as an effective carrier for exosomes, ensuring high retention within the TBI lesion and enabling sustained release of exosomes; 3) practically, the hydrogel perfectly combines injectability, degradability, and biocompatibility. Finally, iNSC‐Exo delivered by our novel injectable hydrogel exhibits therapeutic effects comparable to those of NSC‐Exo, positioning it as a promising alternative for TBI treatment.

Systemic administration of iNSC‐Exo was adopted through venous injection in this study. Indeed, exosomes contain numerous molecules, including proteins, glycoconjugates, lipids, nucleic acids, metabolites, and other bioactive substances.^[^ ^42^, ^43^ ^]^ Exosomes can be surface‐modified to broaden, change, or improve their therapeutic effects, which is exemplified by genetic engineering of exosomal membrane or parental cells, chemical connection of targeting ligands, electrostatic interaction, and magnetic nanoparticle technology.^[^ ^44^ ^]^ To improve the delivery of exosomal cargo to the CNS for enhanced therapeutic effects, the CNS‐specific targeting ligand, RVG peptide, has recently been used to modify the surface of exosomes. This surface modification strategy could be achieved either before or after the exosomes are generated. In this respect, Alvarez‐Erviti et al. modified parental cells using genetic engineering to express Lamp2b and RVG peptides, thereby delivering short interfering RNA to neurons, microglia, and oligodendrocytes in the brain.^[^ ^45^ ^]^ In contrast, Cui et al. conjugated RVG peptide to the secreted exosomes, which exhibited improved targeting to the cortex and hippocampus after being injected intravenously.^[^ ^19^ ^]^ The non‐invasive delivery of our RVG‐iNSC‐Exo has proven to be a superior therapeutic agent to unmodified exosomes, which can significantly improve behavioral outcomes and enhance learning and memory abilities after TBI.

Importantly, the present study presented a comprehensive single‐cell characterization of the peri‐injury brain in TBI mice following iNSC‐Exo treatment. Our single‐cell RNA sequencing analysis revealed that microglia exhibited the most pronounced response to iNSC‐Exo treatment compared to other cell types such as neurons, oligodendrocytes, pericytes, and astrocytes, based on the single‐cell sequencing results. This is consistent with the current understanding within the TBI field, where microglia, the resident immune cells of the CNS, play a crucial but diverse role in the early response (e.g., neuroprotection) and late response (e.g., pro‐neuroinflammation) after TBI.^[^ ^46^ ^]^ Besides, an increase in the abundance of specific microglial subpopulations was observed following TBI and subsequent treatment. This finding may be attributed to the functional disparities of microglia in physiological state (i.e., uninjured brain) and pathological state (i.e., TBI brain), which are intricately linked to their molecular characteristics.^[^ ^47^ ^]^ Most importantly, using single‐nucleus transcriptomics to elucidate the dynamic evolutionary landscape of microglial subpopulations pre‐ and post‐iNSC‐Exo treatment in the context of TBI, we identified two microglial subpopulations, designated as Microglia_Nrg3 and Microglia_Rarb, both of which demonstrated a similar low‐differentiation, stem‐like state. Therefore, our findings move beyond the conventional M1/M2 paradigm, demonstrating a clear separation of microglia into distinct cellular subpopulations that feature temporal characteristics after TBI and after iNSC‐Exo treatment. Our single‐cell sequencing data provide valuable insights into not only the symptomatic and pathological progression following TBI but also therapeutic approaches that modify microglial responses to TBI and improve long‐term outcome measures.^[^ ^48^, ^49^ ^]^

Overall, we provide an unprecedented prediction of the signaling pathways regulated during iNSC‐Exo treatment of mice TBI. Our sub‐populational analysis demonstrated significant enrichment in GO terms related to synaptic and axonal signaling functions, notably within the Microglia_Nrg3 and Microglia_Rarb clusters. Previous research has established the involvement of microglia in synapse formation during both developmental and pathological processes, where they modulate neural circuit plasticity and functionality through mechanisms such as synaptic pruning.^[^ ^50^ ^]^ Our finding further substantiates the critical role of microglia in maintaining neural homeostasis and facilitating injury repair.^[^ ^51^ ^]^ Besides, our cellular communication analysis demonstrated that iNSC‐Exo treatment could markedly enhance the interaction between microglia and neurons, potentially contributing to synaptic recovery post‐TBI in murine models. This effect is potentially facilitated by enhancing the interaction between microglial NRXN with neuronal synaptic proteins, such as Nlgn1. Recent evidence has indicated that activated microglia may contribute to neuronal protection in the adult brain by migrating to inhibitory synapses and physically detaching them from cortical neurons.^[^ ^52^ ^]^ Finally, NRXN, an integral cell surface protein, is essential for the maintenance of synaptic function through its interactions with various synaptic proteins.^[^ ^53^ ^]^ Recent studies have elucidated the critical role of Nrxn1 expressed by microglia in the development and modulation of neural networks,^[^ ^54^ ^]^ and that upregulating neurexins in NSCs offers therapeutic potential for enhancing synaptic regeneration after neural injury.^[^ ^55^ ^]^ In conclusion, iNSC‐Exo treatment of TBI may facilitate the involvement of microglia in neural synaptic regeneration via multiple pathways.

## 4 Conclusion and Future Perspectives

This is the first comprehensive study investigating the therapeutic potential, versatile delivery, and underlying mechanism of iNSC‐Exo treatment of traumatic brain injury. This preclinical study, using a murine model, was designed to simulate the clinical practice during severity‐based TBI management. For severe cases of TBI, exosomes can be locally delivered via a decompressive craniotomy. This intraoperative measure can be further enhanced with a novel injectable hydrogel for sustained, protected release of iNSC‐Exo, which ameliorates brain edema, restores blood–brain barrier integrity, and exerts anti‐neuroinflammatory and neuroprotective effects in TBI mice. For milder cases of TBI, exosomes could be systemically administered through venous injection. This medical treatment could be further enhanced by modifying the exosomes with rabies viral glycoprotein for targeted delivery to the central nervous system, which improves behavioral outcomes, learning capacity, and cognitive functions in TBI mice. Irrespective of the delivery modality, our single‐cell sequencing analysis revealed that iNSC‐Exo treatment could modulate microglia‐neuron synaptic interactions by enhancing the NRXN signaling, particularly involving Nrxn1‐3 derived from the Microglia_Nrg3 and Microglia_Rarb subpopulations. These hierarchical investigations substantiate that iNSC‐Exo represent a versatile and potent therapeutic platform with clinical potential for both surgical (e.g., as a perioperative adjunct to surgery) and medical (e.g., as a standalone pharmacological agent for short‐ and long‐term management) management of TBI. Future research should utilize large animal models, explore alternative delivery modalities (e.g., intranasal, intrathecal, and intracerebroventricular administration of exosomes), and perform time‐dependent mechanistic analysis to account for microglia's dual role in the acute and chronic phases of TBI and its treatment.

## 5 Experimental Section

### Exosome Isolation, Characterization, and RVG‐iNSC‐Exo Formulation

Cortex from C57BL/6 mouse (embryonic day 14) was dissected as previously described.^[^ ^56^ ^]^ Briefly, cortical tissues were separated from fetal brain tissue and filtered through a 40 µm filter. Monodispersed cells were cultured using a neurocult proliferation kit containing human recombinant bFGF, human recombinant EGF, and penicillin‐streptomycin to generate neurospheres. The neurospheres were dissociated and resuspended into monodispersed cells to facilitate the formation of second‐generation neurospheres in suspension culture and subsequent generations using a similar method. All experimental procedures were approved by the Institutional Animal Care and Use Committee at Tongji University (TJAA12124107). To confirm the successful cultivation of neurospheres, immunofluorescence staining was performed using nestin and sex determining region Y‐box 2 (SOX2). The medium was collected (2–6 generations) and centrifuged at 300 g for 10 min, 3000 g for 20 min, and 10 000 g for 30 min. The supernatant was ultracentrifuged (Ultracentrifuge, Beckman Coulter, XPN‐100) at 100 000 g in a 70Ti rotor for 2 h. The pelleted exosomes were called NSC‐derived exosomes (NSC‐Exo) and resuspended in PBS and stored at −80 °C until further use. Induced neural stem cells (iNSC) from mouse fetal foreskin fibroblasts were donated by Zheng Jialin C.’s research group (Tongji University, School of Medicine). Mouse fibroblasts were incubated in the mixed virus‐containing supernatants overnight. After 3 rounds of selection and enrichment, iNSC were collected and generated neurospheres.^[^ ^57^ ^]^ The pelleted exosomes were performed in the same way for the NSC‐Exo as previously described. Each batch of exosomes was quantitatively analyzed using a BCA protein assay kit (Beyotime, China). NSC‐Exo and iNSC‐Exo were characterized by transmission electron microscope (TEM, Talos F200X, FEI, USA), nanoparticle tracking analysis (NTA, Particle Metrix, PMX‐120, Germany), and flow cytometry (FCM, BD Biosciences). The conjugation of RVG to exosomes was carried out by Xiamen Life Interconnection Technology Co., Ltd., following a previously published protocol.^[^ ^58^ ^]^ Briefly, 2 mm PEG‐DESP‐RVG‐FITC and 0.5 mg mL^−1^ iNSC‐Exo in PBS were incubated at room temperature (RT) on a rotating mixer overnight. Unconjugated PEG‐DESP‐RVG‐FITC was removed and washed by two consecutive centrifugation steps at 100 000 g for 70 min at 4 °C. RVG‐iNSC‐Exo was collected and resuspended in PBS and stored at −80 °C for future use. RVG labeling efficiency was detected by nanoflow cytometry (N30E, Xiamen fuliu).

### In Vitro Cellular Uptake and In Vivo Exosomes Tracking

Microglia cells (BV2) were cultured in DMEM containing 10% FBS and 1% penicillin‐streptomycin. The rat pheochromocytoma cells (PC12) were cultured in RPMI‐1640 containing 10% FBS and 1% penicillin‐streptomycin. The exosomes solution (NSC‐Exo, iNSC‐Exo, 0.2 mL, Protein content: 2 mg mL^−1^) was gently mixed with PKH26 diluent solution (1 mL, Solarbio, CatD0030) and incubated for 30 min in the dark at 4 °C. PBS (20 mL) was added to the mixture and ultracentrifuged at 100 000 g for 2 h at 4 °C, and the supernatant containing unbound dye was discarded. The labeled exosomes (NSC‐Exo and iNSC‐Exo; protein content: 10 µg) were incubated with BV2 cells or PC12 cells (4 × 10⁴ cells well^−1^) at 37 °C. After 24 and 48 h, fluorescence was observed using a laser confocal microscope (Nikon, Japan), and nuclei were stained with DAPI. In a separate set of experiments, the labeled exosomes (iNSC‐Exo; protein content: 10, 20, 30 µg) were incubated with BV2 cells or PC12 cells (4 × 10⁴ cells well^−1^) at 37 °C. After 48 h, fluorescence was observed using a laser confocal microscope, and nuclei were stained with DAPI. Controlled cortical impact (CCI) injury was a widely utilized method for establishing TBI models. In this study, C57BL/6 male mice (8 weeks) were subjected to CCI injury routinely. The mice were anesthetized with 1.25% tribromoethanol (0.02 mL g^−1^; Nanjing AiBi Bio‐Technology Co. Ltd.). Following skin antisepsis and sterilization, a 4 mm skull window was created using a sander drill (AGD, USA). The injury was induced with the aid of a stereotaxic frame (Kopf Instruments, Tujunga, CA, USA) and a stereotaxic impactor (Leica Biosystems Richmond, USA). To ensure precision, the angle between the exposed cortex and the impactor was carefully adjusted. Then, the mice were subjected to impact (deformation, 0.5 mm; piston velocity, 3.05 m s^−1^). The exosomes were labelled with DiD perchlorate (MedChem Express, US) for the biodistribution detection. TBI mice were given intravenous administration (IV) of DiD‐labeled iNSC‐Exo (200 µL mouse^−1^), DiD‐labeled RVG‐iNSC‐Exo (200 µL mouse^−1^), or PBS. The distribution of DiD‐labeled exosomes was observed and quantification by in vivo fluorescence imaging at different time points using an in vivo optical imager (VISQUE in Vivo Smart‐LF, Korea). On 24, 48, and 96 h, the mice were sacrificed, and their major organs (heart, liver, spleen, lungs, and kidneys) and brain were harvested for further fluorescence determination of DiD (MCE, HY‐D1028). Brain tissues were fixed with 4% paraformaldehyde, sectioned, and immunofluorescence staining with ionized calcium‐binding adapter molecule 1 (Iba‐1) and DAPI. Slices were imaged and detected DiD, FITC (RVG), Iba‐1(Proteintech, 10904‐1‐AP), and DAPI (Sigma‐Aldrich, MBD0015) signals in brain tissue.

### Cell Experimental Protocol

BV2 cells were seeded evenly in 24‐well plates at a density of 1.5 × 10⁵ cells well^−1^ and incubated for 24 h. The cells were then co‐incubated with lipopolysaccharide (LPS, Beyotime Biotechnology, 500 ng mL^−1^). Then, the treated cells were co‐incubated with different exosomes for 24 h. NSC‐Exo (100 µg mL^−1^) and iNSC‐Exo (100 µg mL^−1^) were used as experimental groups in the presence of LPS. The LPS group received LPS (500 ng mL^−1^) alone to induce inflammation, while untreated BV2 cells served as the control group. The in vitro experimental procedure is shown in Figure S17 (Supporting Information). Cells were collected from each group for Quantitative real‐time PCR (Q‐PCR). Total RNA was extracted from BV2 cells using an RNA quick purification kit (ES Science, China), and 2 µg of total RNA was used for reverse transcription with reverse transcriptase agents (Vazyme, China) to synthesize cDNA (Table S1, Supporting Information). Q‐PCR was performed using SYBR Master mix agents (Vazyme, China) with an RT‐PCR system (QuantStudio 7 Flex, Thermo Fisher, USA). To calculate the relative expression levels of mRNA, the 2^−ΔΔCt^ method was used. GenScript Biotech (Nanjing, China) synthesized the primers, and the primer sequences are listed in Table S1 (Supporting Information). After treatment by different groups, the BV2 cells were stained with anti‐TNF‐*α*‐PE and anti‐IL4‐ Qdot 605 for 2 h in the dark, washed, resuspended in PBS, and analyzed using a FACS system (BD Biosciences).

The PC12 cells were cultured in RPMI‐1640 containing 10% FBS and 1% penicillin‐streptomycin. PC12 cells were seeded evenly in 24‐well plates at a density of 5 × 10^4^ cells well^−1^ and then placed in an incubator with CO_2_ for 24 h. A longitudinal scratch was made in each well to create a TBI cell model when the cells attained a confluence of 80–90%. The scraped cells were rinsed with PBS. Then, the cells were treated with NSC‐Exo (100 µg mL^−1^) and iNSC‐Exo (100 µg mL^−1^) for 24 h. Cells were collected from each group for Q‐PCR according to the above method described. In a separate set of experiments, a scratch injury of a wide gap was made with a 10 µL plastic stylet needle. The scraped cells were rinsed with PBS. Then, the cells were treated with NSC‐Exo (100 µg mL^−1^) and iNSC‐Exo (100 µg mL^−1^) for 24 h. Immunofluorescence staining (growth‐associated protein, GAP‐43) of PC12 cells was carried out. Prior to immunofluorescence staining, the cells were fixed with 4% paraformaldehyde (PFA) for 10 min and permeabilized with 0.2% Triton X‐100 for 10 min. Then, the cells were blocked for 1 h with blocking solution (1% goat serum in PBS). The primary antibodies (GAP‐43 polyclonal antibody, Proteintech) were diluted 1:100 in PBS and incubated with cells for 24 h at 4 °C. The next day, the cells were incubated secondary antibodies (Alexa Fluor488, Beyotime) for 1 h at room temperature. The cells were examined under a fluorescence microscope (CKX53; Olympus) after counterstaining with DAPI. Cells with axons whose size was greater than or equal to the length of the cell body were identified as positive cells. Cells in different random views were counted for each well (*n* = 50). The neurites in each cell were traced with the mouse using the Image J program software, and the neurite length was calculated.

### Injectable Hydrogel Synthesis and Characterization

Carboxymethyl chitosan solution (2% w/v) was prepared by dissolving 1 g of carboxymethyl chitosan (CMCS, Taitan) in 50 mL PBS, and centrifuged to remove any undissolved materials. Sodium alginate (SA, Taitan) was stirred in PBS to form a SA solution with a concentration of 2% (w/v). CMCS and SA solution (Mixture) were mixed and stirred for 1 h while keeping the total mass of CMCS and SA constant. *D*‐gluconic acid *δ*‐lactone (GDL, Aladdin) was stirred in PBS to form a GDL solution with 2% (w/v). The solutions were then mixed in a Mixture and allowed to gel (Hydrogel). The hydrogel precursor containing 1% Rhodamine B dye was placed in a syringe, injected into a bottle or mold, and incubated at 37 °C to allow hydrogel formation. Additionally, the different derived exosomes (Milk, NSC, iNSCs, 1 mg g^−1^) were encapsulated into hydrogels, denoted Milk‐Exo@Gel, NSC‐Exo@Gel, and iNSC‐Exo@Gel. The hydrogel sample was lyophilized and sputter‐coated with gold. The microstructures were examined using a field emission scanning electron microscopy (FE‐SEM, Sigma 300, ZEISS, Germany). Thermal gravimetric (TG) and differential scanning calorimeter (DSC) analysis were recorded on a Pyris1 TGA instrument (PerkinElmer) and DSC instrument (DSC3+, Mettler Toledo). Attenuated total reflectance Fourier transform infrared spectrometer was carried out on a FTIR spectrophotometer (Nicolet I N10) at a range from 500 to 4000 cm^−1^ and a resolution of 2 cm^−1^. The rheological characterization of the hydrogel was conducted on a rheometer (ARES‐G2, TA instruments). Determination of exosome release kinetics in hydrogel. Milk‐Exo@Gel was incubated in PBS at 37 °C, and the supernatant was collected at days 1, 2, 3, 7, 14, 21, and 28. At each time point, the collected supernatant was completely replaced with fresh PBS. The concentration of Milk‐Exo in the supernatants was quantified using the BCA protein concentration assay kit. Additionally, a blank hydrogel was detected as a substrate control to correct the effect of hydrogel degradation in the system.

### Retention Time and Degradation Studies In Vivo

Retention time was evaluated by intra‐operative implantation into the injury cavity. DiR‐labeled Milk‐Exo were loaded into the hydrogel according to the same method as Milk‐Exo@Gel. DiR‐labeled Milk‐Exo@Gel was injected into the injury cavity of TBI mice, and those mice were imaged using an in vivo imaging system (Berthold, GER) to measure the fluorescence intensity of DiR at 0, 7, 14, 21, and 28 days. DiR‐labeled Milk‐Exo (200 µL) was injected into the injury cavity as a control. For the assessment of hydrogel degradation in vivo, the mice were anesthetized with 1.25% tribromoethanol (0.02 mL g^−1^; Nanjing AiBi Bio‐Technology Co. Ltd.). Following skin antisepsis and making an incision, a 4 mm skull window was created using a sander drill (AGD, USA). Hydrogel was implanted into the CCI surgical area (without injury) of C57BL/6 mice. At the defined intervals, the hydrogel was taken out, washed with PBS, and its residual weight was measured. The biocompatibility of the hydrogel was determined by hematoxylin and eosin (H&E) staining of the surrounding brain tissue at different time intervals after implantation.

### Neuroprotection in TBI Mice through iNSC‐Exo@Gel

C57BL/6 (8 weeks, male) were subjected to the CCI injury. The mice were randomized into 4 groups (*n* = 6–9): control, TBI, NSC‐Exo@Gel, and iNSC‐Exo@Gel groups. Cerebral edema was determined by measuring the brain water content at 72 h after TBI. Following anesthesia and decapitation, mice were sacrificed, and the brains were collected immediately. The injured brain specimens were weighed to obtain the wet weight. Then the brain specimens were dried in an oven and weighed again for dry weight. The brain water content was calculated as following formula:

(1) $$\begin{array}{ccc} & & \mathrm{Brain}\;\mathrm{water}\;\mathrm{content}\;\left(\%\right)\\ & & \;=\left(\mathrm{Wetw}\mathrm{eight}\;-\;\mathrm{Dryw}\mathrm{eight}\right)/\left(\mathrm{Wetw}\mathrm{eight}\right)\;\times \;100\% \end{array}$$

BBB permeability was evaluated by measuring the extravasation of EB dye in brain tissue 7 days post‐TBI injury. Mice were anesthetized 24 h after injection of EB dye (2%, 4 mL Kg^−1^) and were infused with PBS through the heart for sufficient elimination of the intravascularly localized dye. The injured brain specimens were weighed to obtain the wet weight. Each sample was soaked in methanamide solution and homogenized (200 mg mL^−1^, 48 h) and then centrifuged at 5000 rpm for 15 min, and the supernatant was transferred. A microplate reader (Tecan, infinite F50, Austria) was used to detect the absorbance of the mixture at 620 nm. The quantity of dye referenced the standard curve.

Residual motor dysfunction was evaluated using the rotarod and balance beam tests. The rotarod tests were performed by mouse rotating rod fatigue meter (RWD Life Science Co., Ltd). Briefly, the mice were placed on the wooden beam and subjected to balance beam training 3 days before CCI. On days 3, 7, 14, 21, and 28 following TBI, the test was performed. The total step number, the missed step number, and the missed step ratio were recorded and calculated. The rotarod tests were performed via a mouse rotating rod fatigue meter (RWD Life Science Co., Ltd). Training of the mice was conducted 3 days prior to CCI, and the test was performed on days 1, 3, 7, 14, 21, and 28 after TBI. The residence times on the rod were recorded, and the maximum retention time is 300 s. Brain tissues were harvested from the mice after 28 days following TBI. The brain tissues were fixed with 4% paraformaldehyde, sectioned, and stained with H&E, and immunofluorescence stained including GFAP, Iba‐1, Doublecortin‐like kinase 1 (DCX), and GAP‐43. On the 28th day, blood samples were collected for biochemical analysis (Sevilla Biotechnology Co., Ltd.). On the 28th day, the main organs (heart, liver, spleen, lung, and kidney) were harvested. Then, the tissues were fixed with 4% paraformaldehyde, sectioned, and stained with H&E.

### Evaluation of Neurological Function by RVG‐iNSC‐Exo System Administration

C57BL/6 (8 weeks, male) were subjected to the CCI injury. The mice were randomized into 4 groups (*n* = 6–9): control, TBI, iNSC‐Exo, and RVG‐iNSC‐Exo groups. Each mouse received 10 µg g^−1^ exosomes through IV (Day 1, 3, 5, 7, and 9) except the TBI group and the control group. The TBI group and control group received saline through IV. Residual motor dysfunction was evaluated using the rotarod and wire grip tests. Training of the mice was conducted 3 days prior to CCI, and the test was performed on days 1, 3, 7, and 14 after TBI. The residence times on the rod were recorded, and the maximum retention time is 300 s. For the hanging wire test, mice were briefly held by their forelimbs on a wire (50 cm in length and 0.3 cm in diameter) strung between two points, positioned 45 cm above a layer of bubble wrap. Each mouse underwent three trials, with scores assigned according to their performance within 1 min (Table S2, Supporting Information). The inter‐trial intervals for all subjects were 20 min. The Morris water maze test was conducted to evaluate the cognitive learning capacity and spatial memory of mice. The test was conducted 15 to 20 days following TBI. Mice were placed in a circular tank filled with water, which was divided into four equal quadrants. Visual indicators within the visual field of the mouse were positioned around the pool to denote the submerged platform. In each trial, the mouse was given a maximum of 60 s to locate the submerged platform before being directed to it, taken out of the water, towel‐dried, and returned to its cage. Each mouse undertook 4 trials daily throughout the 5 days training period. Multiple characteristics of mouse motility were documented, including the searching strategies and the time required for climbing the platform (escape latency). The probe test was administered one day following the training. The platform was taken away, and each mouse was allotted 60 s to swim in the water. The duration of residence and the number of platform crossings in each quadrant were documented by a computer.

For electrophysiological experiments, mice were anesthetized with isoflurane gas via a circulating anesthesia system and secured in a stereotaxic apparatus with the head positioned horizontally. Using the bregma and lambda as reference points, a hole was drilled at the target location (CA1 region: −2 mm AP, +1.7 mm ML, −2.7 mm DV) based on the mouse brain atlas. After exposing the skull and confirming the coordinates, A 4 × 2 electrode array with 8 microwires was carefully inserted into the target brain region to record LFP. The electrode was fixed to the skull surface with dental acrylic to ensure stability and prevent postoperative displacement. Postoperative care was provided to maintain the physiological condition of the experimental animals. On the 22nd day, the brain tissues were harvested and photos were taken. The main organs (heart, liver, spleen, lung, kidney) were harvested. Then, the tissues were fixed with 4% paraformaldehyde, sectioned, and stained with H&E. Brain tissues were subjected to H&E staining followed by quantitative analysis of lesion area in each group. Blood samples were collected from all experimental groups for measurement of hepatic and renal biochemical parameters.

### Whole‐Transcriptome Amplification Single‐Cell RNA Sequencing

Fresh tissue samples were dissociated and digested to obtain single‐cell suspensions (or nuclei suspensions for frozen or specialized tissues). The resulting single‐cell (or nuclei) suspensions were washed and resuspended in appropriate buffer solutions. Quality control (QC) was performed to ensure the following criteria were met: cell viability ≥ 80%, live cell concentration ranging from 700 to 1200 cells µL^−1^, and cell diameter within the range of 5–40 µm. These suspensions were then prepared for single‐cell capture using the 10× Genomics Chromium platform.

Single‐cell capture was performed using the 10x Genomics Chromium platform, which employed microfluidic technology to encapsulate individual cells, gel beads containing cell barcodes and primers, and lysis reagents into oil droplets, forming “micro‐reactors”. Within each micro‐reactor, single cells were lysed to release RNA, which was then hybridized to the barcoded primers on the gel beads. Reverse transcription was subsequently carried out to generate barcoded cDNA for downstream sequencing.

The oil droplets were broken to release the barcoded cDNA, which was then purified and enriched using magnetic beads. The cDNA was amplified via polymerase chain reaction (PCR) and subjected to quality control to ensure its suitability for library construction.

The amplified cDNA was fragmented, and sequencing adapters and indices were ligated to construct next‐generation sequencing libraries. The quality of the constructed libraries was assessed using appropriate QC metrics, including fragment size distribution and concentration measurements.

Sequencing was performed on the Illumina sequencing platform (compatible with MGI T7 platform) using paired‐end 150 bp (PE150) mode. A recommended sequencing depth of 100 Gb was achieved, corresponding to ≈30 000 read pairs per cell. Additional sequencing was conducted if necessary, depending on the specific experimental requirements and data quality. The raw sequencing data were processed using the 10× Genomics official analysis pipeline, Cell Ranger (https://support.10xgenomics.com/single‐cell‐gene‐expression/software/overview/welcome), to perform data filtering, alignment, quantification, and cell identification. This pipeline generated a gene expression matrix for each individual cell. In cases where batch effects were observed, the Seurat RPCA (Reciprocal Principal Component Analysis) method was employed to correct for batch effects. Potential doublets or multiplets were predicted and removed using Scrublet to ensure data quality. The gene expression matrix was subsequently subjected to dimensionality reduction and clustering analysis using principal component analysis (PCA) and t‐SNE/UMAP algorithms. Cell clustering was performed using Seurat, and the results were visualized graphically. Differential gene expression analysis was conducted across different clusters, and the results were presented using violin plots, t‐SNE/UMAP plots, and cluster heatmaps. Top differentially expressed genes (DEGs) identified in each cluster were visualized using volcano plots and feature plots. Functional enrichment analysis of the DEGs was performed, including GO, KEGG, and Reactome pathway analysis (limited to human and mouse data). Cell type annotation was performed based on provided marker genes or using the SingleR package for automated cell type identification.

### Analysis of Differentially Expressed Genes (DEGs) and Functional Pathways Enrichment

Differential expression analysis of single‐cell RNA sequencing (scRNA‐seq) data was performed using the Seurat package (Version 4.4.0).^[^ ^59^ ^]^ Cells labeled as “iNSC‐Exo” and “TBI” were analyzed across different subtypes (sub_celltype). DEGs were identified using the FindMarkers function, with significance thresholds set at adjusted *p‐*value (*p*_val_adj) < 0.05 and |avg_log2FC| ≥ 0.58. Genes meeting these criteria were classified as either upregulated or downregulated.

Cell types most relevant to one specific group were ranked by the number of DEGs. Cell type prioritization analysis under three groups (Control, iNSC‐Exo, and TBI) was done using the Augur R package (version 1.0.3).^[^ ^60^ ^]^

To gain insights into the biological functions associated with DEGs in each cell subtype, GO and KEGG pathway enrichment analyses were performed using Metascape^[^ ^61^ ^]^ (https://metascape.org). Upregulated and downregulated DEGs were analyzed separately for each cell subtype.

For GO and KEGG enrichment analysis, significantly enriched terms were identified based on *P* < 0.01. To ensure biological relevance, the top five overlapping pathways were selected among different subtypes for visualization.

### Gene Set Variation Analysis

GO and KEGG pathway gene sets were retrieved from the molecular signatures database (MSigDB). To assess pathway activity at the single‐cell level, pathway activity scores for each cell were computed using GSVA.^[^ ^62^ ^]^ To identify pathways with differential activity between groups, Wilcoxon rank‐sum tests were performed for each subtype.

### Pseudotime and Trajectory Analysis and Cytotrace

Pseudotime and trajectory analysis were conducted utilizing Monocle software (version 2.32.0) with default parameters. Microglial subpopulations were examined to infer the pseudotime trajectory. Group‐specific marker genes were identified through the application of the “detectGenes” function. The DDRTree algorithm was used for dimensional reduction. Subsequently, cells were pseudo‐temporally ordered employing the “reduceDimension” and “orderCells” functions. The expression dynamics along the trajectories were visualized using the BEAM function. The “differentialGeneTest” function was used to calculate pseudo‐temporal dependent DEGs, and the pseudo‐temporal expression heatmap was demonstrated by “plot_pseudotime_heatmap”.

The single‐cell RNA sequencing data were subsequently analyzed utilizing CytoTRACE (https://cytotrace.stanford.edu), an algorithm that facilitates the robust reconstruction of cellular differentiation trajectories, in order to trace dynamic chromatin changes within the mouse brain microglia dataset.

### Cell–Cell Ligand‐Receptor Communication Analysis

Cell–cell communication networks between microglia and neurons were inferred and visualized using the CellChat (Version 1.6.1) R package,^[^ ^63^ ^]^ which systematically analyzed scRNA‐seq data to identify ligand–receptor interactions. The CellChat database comprised 1939 curated ligand–receptor pairs, including 1199 secreted signaling interactions, 319 cell–cell contact interactions, and 421 extracellular matrix–receptor interactions. To compare intercellular communication across conditions, interaction counts and interaction strengths were quantified between groups and assessed differences in the global communication patterns of individual signaling pathways by comparing overall information flow. A minimum threshold of 10 cells per cell group was applied for robust inference of cell–cell communication. Significant ligand–receptor interactions with *P* < 0.05 were extracted for downstream analysis. For each identified ligand–receptor pair, violin plots were generated to visualize expression levels across different groups and samples.

### Transcription Factor Regulatory Network Analysis (SCENIC)

Single‐cell regulatory network inference and clustering (SCENIC) was a computational framework designed to reconstruct transcriptional regulatory networks from scRNA‐seq data. SCENIC consisted of three key steps: co‐expression analysis, motif enrichment analysis, and regulon activity evaluation.

In the first step, GENIE3 was employed to infer co‐expression modules by predicting associations between transcription factors (TFs) and candidate target genes. Each module comprised a TF and its putative target genes, identified solely based on co‐expression patterns.

In the second step, RcisTarget was used to assess whether the predicted target genes of each module were significantly enriched for known TF‐binding motifs. Only modules with statistically enriched TF motifs were retained, ensuring the identification of high‐confidence regulons—defined as TFs and their directly regulated target genes.

In the third step, AUCell quantified the activity of each regulon at the single‐cell level. AUCell computed the area under the recovery curve (AUC) for the genes within each regulon, generating a regulon activity matrix. This matrix could be binarized by applying an AUC threshold to define whether a regulon is “active” in a given cell. The resulting regulon activity profiles enabled the clustering of cells based on shared transcriptional regulatory programs, facilitating the identification of distinct cell types and states.

Using the SCENIC (Version 1.3.1) R package,^[^ ^64^ ^]^ transcription factor (TF) enrichment and regulon activity could be analyzed across different cell populations. Furthermore, Cytoscape was utilized to construct regulatory networks, visualizing enriched TFs and their associated target genes.

### Immunofluorescence Staining of Mouse Brain Tissue and Statistical Classification of Intercellular Distances

Paraffin‐embedded brain sections (5 µm) from control, TBI, and iNSC‐Exo groups (*n* = 3/group) were subjected to multiplex immunofluorescence staining. After standard deparaffinization and antigen retrieval (citrate buffer, pH 6.0), sections were incubated with primary antibodies against NeuN (1:300, Abcam, ab104224), Nrg3 (1:200, Bioss, bs‐6235R), and Rarb (1:300, Proteintech, 14013‐1‐AP) overnight at 4 °C. Appropriate Alexa Fluor‐conjugated secondary antibodies (1:1000, Invitrogen) were applied for 2 h at room temperature. Nuclei were counterstained with DAPI (Sigma‐Aldrich, MBD0015). The scanning of the tissue slices was performed with a high‐resolution digital slide scanner, the Pannoramic MIDI by 3DHistech. Target microglial populations were defined as Nrg3+/NeuN‐ or Rarb+/NeuN‐ cells, while neurons were identified as NeuN+ cells with characteristic morphology. Two non‐adjacent sections per animal were analyzed, with 1 field of interest per section. The distance calculation and cell pairing were performed using a “nearest‐neighbor priority matching with deduplication” approach. This method ensured that: 1) each target cell (Nrg3+/NeuN‐ or Rarb+/NeuN‐) was paired with its nearest NeuN+ neuron within a 200 µm radius; 2) when multiple target cells matched to the same neuron, only the closest pair was retained to avoid duplicate counting; and 3) unmatched cells beyond the 200 µm threshold were excluded from subsequent proportional distribution analysis. Image processing was conducted using Image J. The individual color channels were isolated, and cellular identification was performed as follows: red cells were defined by the intersection of the red and blue channels while excluding the green channel, whereas green cells were identified as the overlapping regions of the green and blue channels. Intercellular distance measurements were computed using a custom MATLAB (R2023b) algorithm based on the distance calculation and cell pairing approach described above and the results were analyzed for each image.

### Statistical Analysis

Statistical analyses and data visualizations were conducted using GraphPad Prism 10.0 (GraphPad Software, La Jolla, CA, USA), MATLAB (MATLAB R2023b), and Origin 9.0. All mean values are presented as mean ± SD. Statistical differences between multiple groups were analyzed by one‐way ANOVA, two‐way ANOVA, or two‐way repeated‐measures ANOVA followed by Tukey's multiple‐comparisons test. The detailed statistical analysis, such as the sample size (*n*) for each analysis could also be accessed in the figure legend. Asterisks denote corresponding statistical significance: *P < 0.05, **P < 0.01, ***P < 0.001.

## Conflict of Interest

The authors declare no conflict of interest.

## Supporting information



Supporting Information

## Data Availability

The data that support the findings of this study are available from the corresponding author upon reasonable request.
