# Supplementary material for: Clinically Inspired Multimodal Treatment Using Induced Neural Stem Cells‐Derived Exosomes Promotes Recovery of Traumatic Brain Injury through Microglial Modulation
Source: Adv Sci (Weinh). 2025 Sep 24;12(46):e08574. doi: 10.1002/advs.202508574 (PMC12697803; doi:10.1002/advs.202508574)
Supplement: Supplementary file 1 — Supporting Information [file ADVS-12-e08574-s001.docx]

**Supporting Information**

**Clinically Inspired Multimodal Treatment Using Induced Neural Stem Cells-Derived Exosomes Promote Recovery of Traumatic Brain Injury Through Microglial Modulation**

Jiaojiao Li ^1,2,†^, Maoxiang Xu ^1,2,†^, Boyu Cai ^1,2,†^, Xiangyu Li ^3^, Zhanping Liang ^3^, Xiaohuan Xia ^3^, Haitao Zhang ^5^, Zhiwen Zhang ^6^, Fei Tan ^1,2,7,*,†,^^, Jialin Charlie. Zheng ^3,4, ^^

*^1^ Department of ORL-HNS, Shanghai Fourth People’s Hospital Affiliated to Tongji University School of Medicine, Shanghai, 2000434, China*

*^2^ Plasma Medicine and Surgical Implants Center, Tongji University School of Medicine, Shanghai, 200092, China*

*^3^ Center for Translational Neurodegeneration and Regenerative Therapy, Tongji Hospital Affiliated to Tongji University School of Medicine, Shanghai, 200092, China*

*^4^ Shanghai Frontiers Science Center of Nanocatalytic Medicine, Tongji University, Shanghai, 200331, China*

*^5^ Department of Neurosurgery, the Fourth Medical Center of PLA General Hospital, Beijing，100048, China*

*^6^ Department of Neurosurgery, Shanghai Fourth People’s Hospital Affiliated to Tongji University School of Medicine, Shanghai, 2000434, China*

*^7^ Department of ORL-HNS, The Royal College of Surgeons of England, London, WC2A3PE, UK*

* Correspondence: Fei Tan; Tel.: +86 21 55603999; Fax: +86 21 56660851. E-mail address: 、feitan@tongji.edu.cn

† All authors contributed equally as first authors.

^ Both authors contributed equally as corresponding authors.

Table S1. Primer sequences for RT-PCR used in this research

| **Gene Name** | | **Forward** | **Reverse** |
| --- | --- | --- | --- |
| CD86 | TCAATGGGACTGCATATCTGCC | | GCCAAAATACTACCAGCTCACT |
| TNF-α | CCCTCACACTCAGATCATCTTCT | | GCTACGACGTGGGCTACAG |
| iNOS | GGAGTGACGGCAAACATGACT | | TCGATGCACAACTGGGTGAAC |
| CD206 | CTCTGTTCAGCTATTGGACGC | | CGGAATTTCTGGGATTCAGCTTC |
| TGF-β | TCGACATGGATCAGTTTATGCG | | CCCTGGTACTGTTGTAGATGGA |
| IL4 | GGTCTCAACCCCCAGCTAGT | | GCCGATGATCTCTCTCAAGTGAT |
| GAP-43 | GCACATCGGCTTGTTTAGGCT | | GGAGGGAGATGGCTCTGCTACT |
| NF-200 | CGGAGGGAAAGGAAGAAATAAA | | AGAGGGACACCCAGAATAGCC |
| Nestin | CTGAGGCCTCTCTTCTTCCA | | ACTCCTGTACCGGGTCTCCT |
| GAPDH | AGGTCGGTGTGAACGGATTTG | | GGGGTCGTTGATGGCAACA |

Table S2 Hanging wire test

| **Behavior** | **Point** |
| --- | --- |
| Detached within 30 seconds | 0 |
| Clung to the wire with two forepaws | 1 |
| Clung to the wire with two forepaws and one hindpaw | 1.5 |
| Clung to the wire with all four paws, but without the tail wrapped | 2 |
| Clung to the wire with all four paws and the tail wrapped around the wire, remaining stationary | 3 |
| Clung to the wire with all four paws and the tail wrapped, exhibiting slight movement | 4 |
| Clung to the wire with all four paws and the tail wrapped, and moved to one end | 5 |

Table S3. Frequency bands and different states of the brain

| **Name** | **Frequency (Hz)** | **Amplitude**  **(μV)** | **Brain activity** |
| --- | --- | --- | --- |
| δ | 0.5-4 | 20-200 | Deep and unconscious sleep |
| θ | 4-8 | 20-150 | Thinking activities: intuition, creativity, recall and mental effort |
| α | 8-13 | 20-100 | Subconsciousness, relaxed wakefulness |
| β | 13-30 | 5-20 | Mental states: active concentration, task engagement, excitement, anxiety, attention, or vigilance |
| γ | 30-45 | ＜5 | Cognitive function: arousal and perceptual |


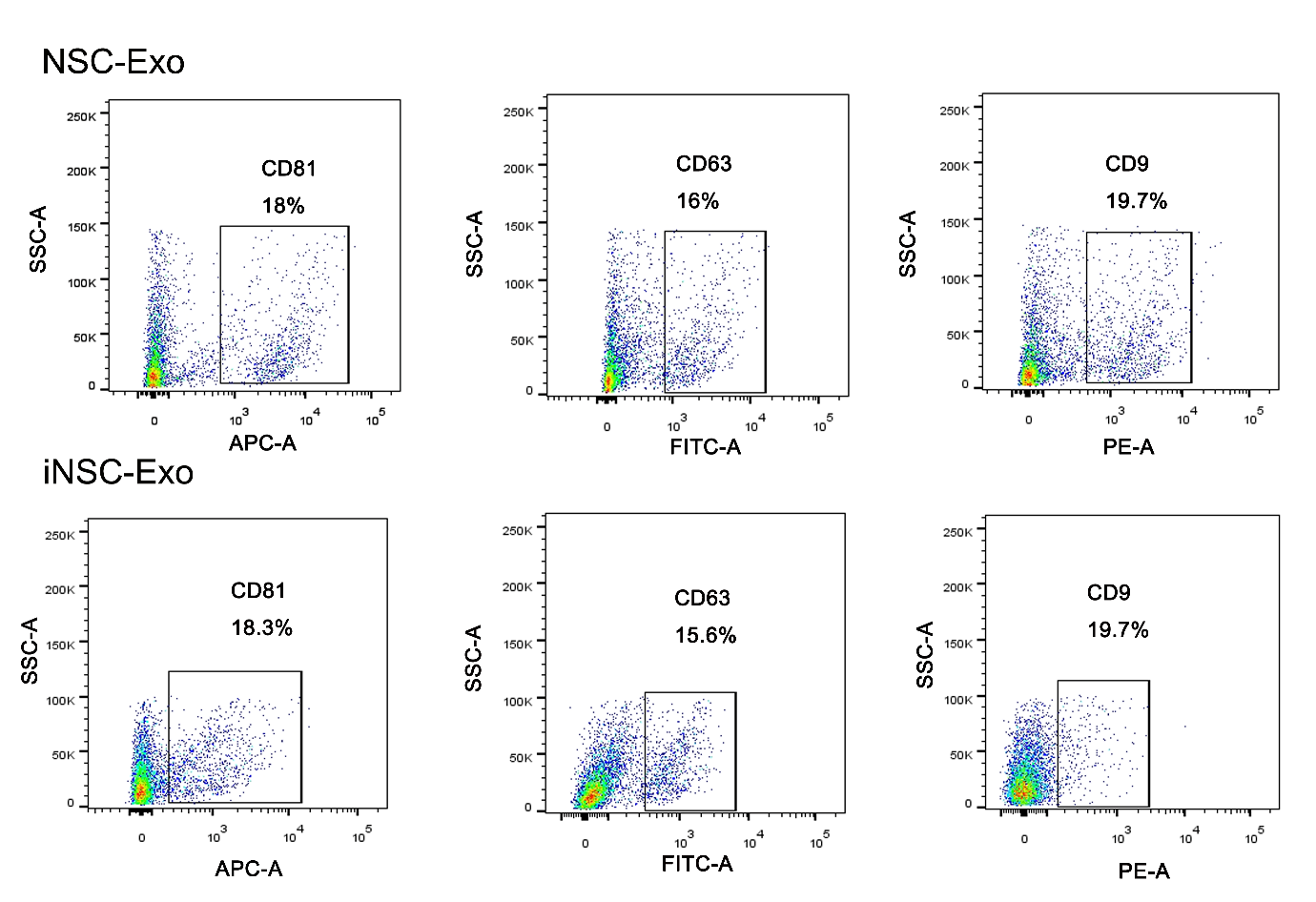


Fig. S1 Exosomal-specific protein markers CD81, CD63, and CD9 investigated by FCM.


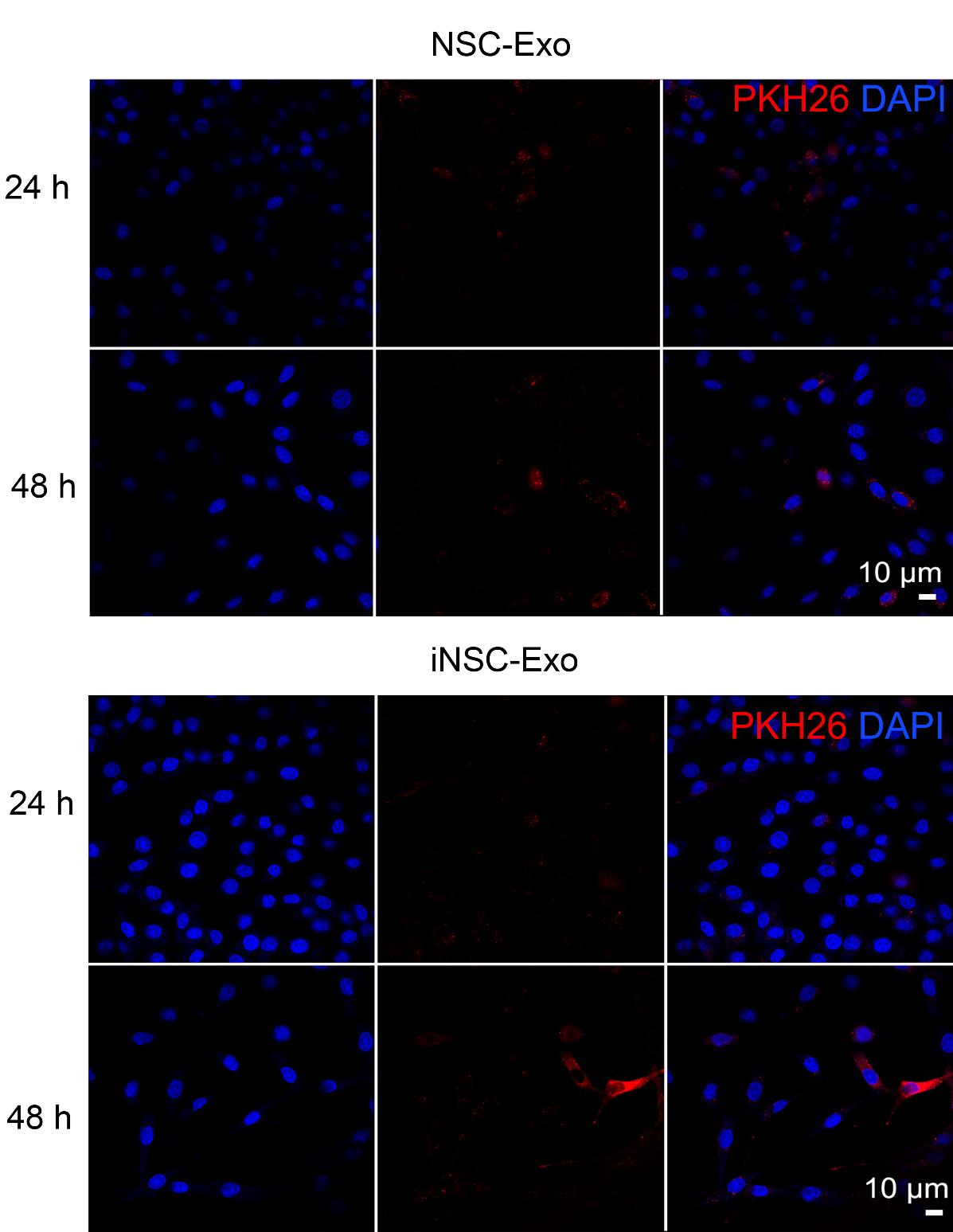


Fig. S2 Internalization of NSC-Exo or iNSC-Exo in PC12 cells. Labelling of exosomes and PC12 cell nucleus by PKH26 (red) and DAPI (blue).


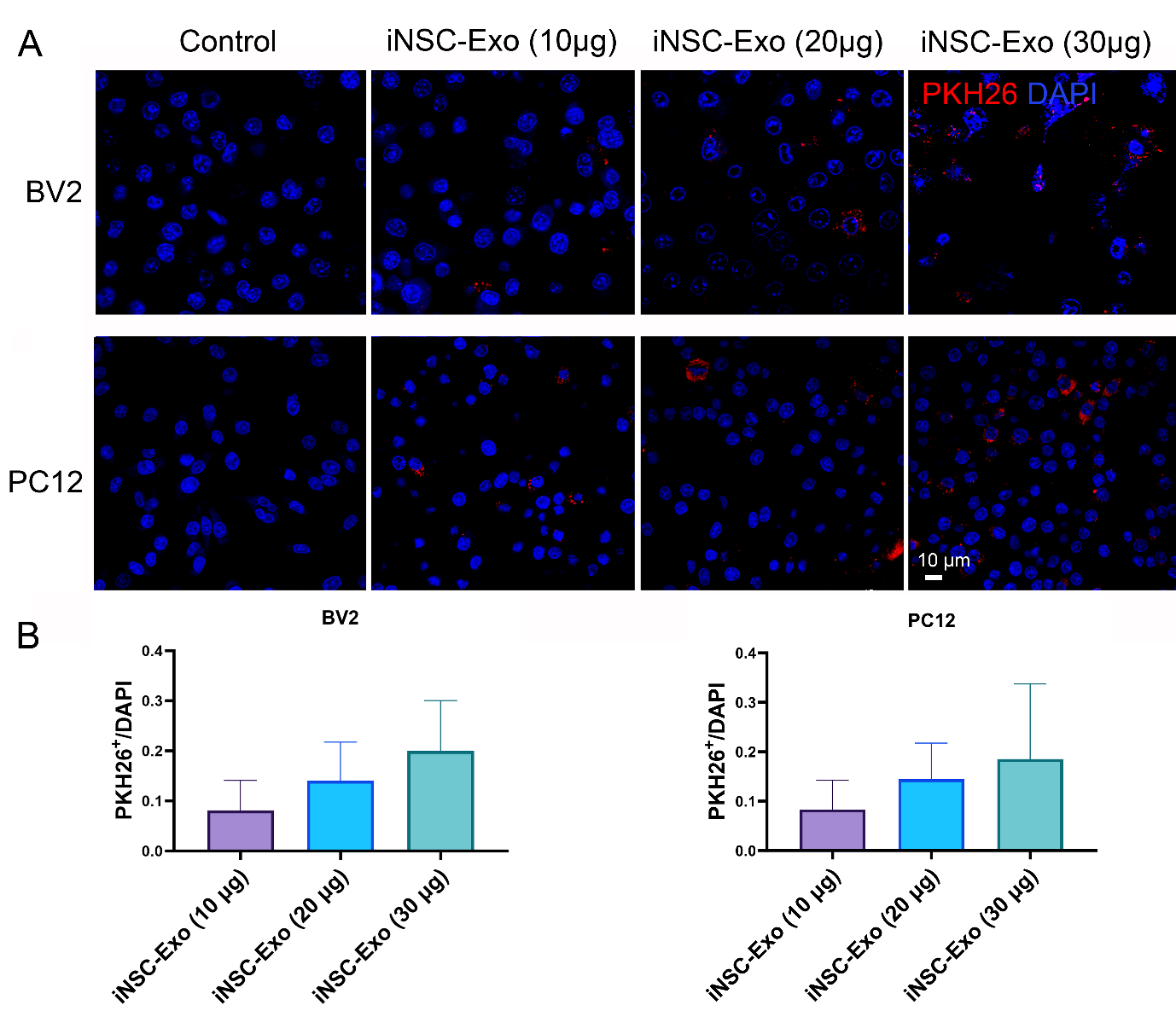


Figure S3 Dose dependence of iNSC-Exo internalization. (A) Representative confocal microscopic images were acquired from cellular areas treated with different doses of exosomes. (B) The relative number of exosomes in each group was quantified as the ratio of PKH26 fluorescence intensity to DAPI fluorescence intensity. Data are presented as the mean ± SD (n=3)


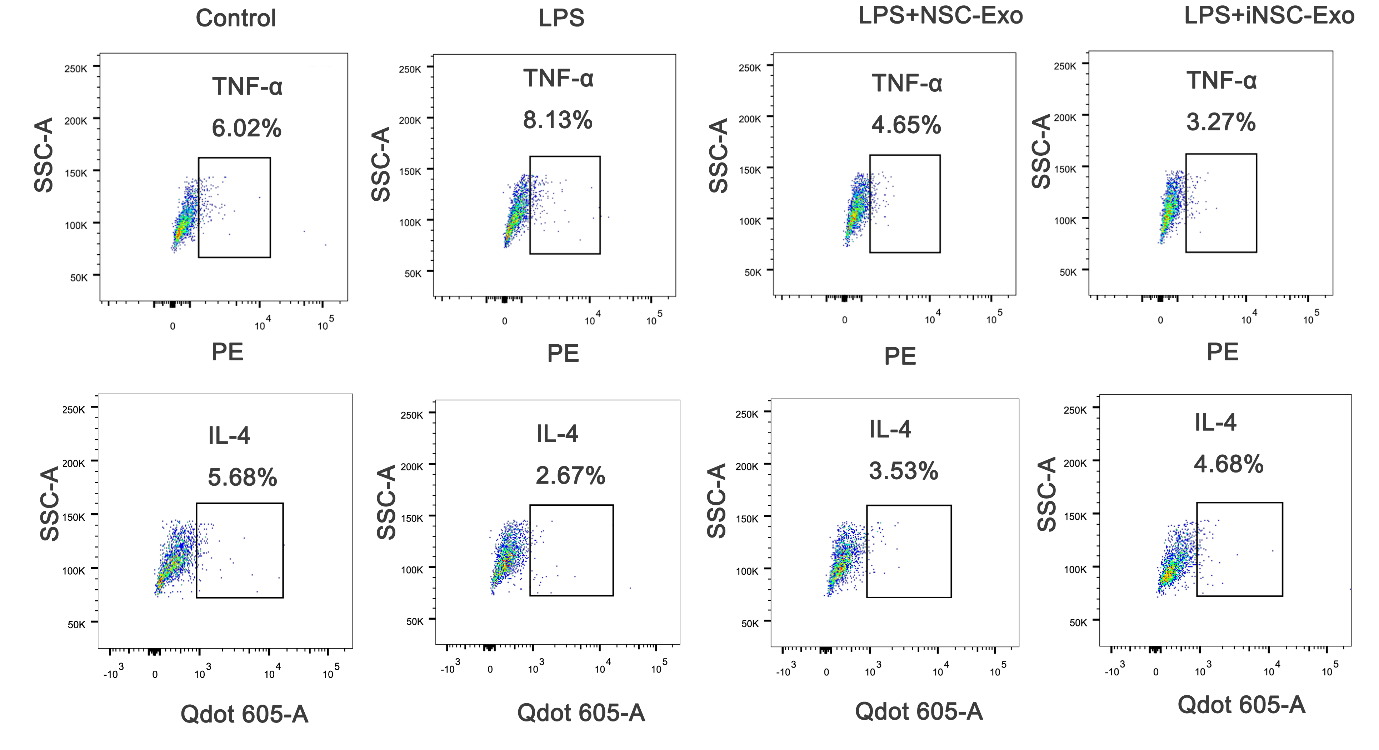
Fig. S4 Representative FCM images of BV2 cells treated with PBS, LPS, LPS+NSC-Exo, and LPS+ iNSC-Exo.


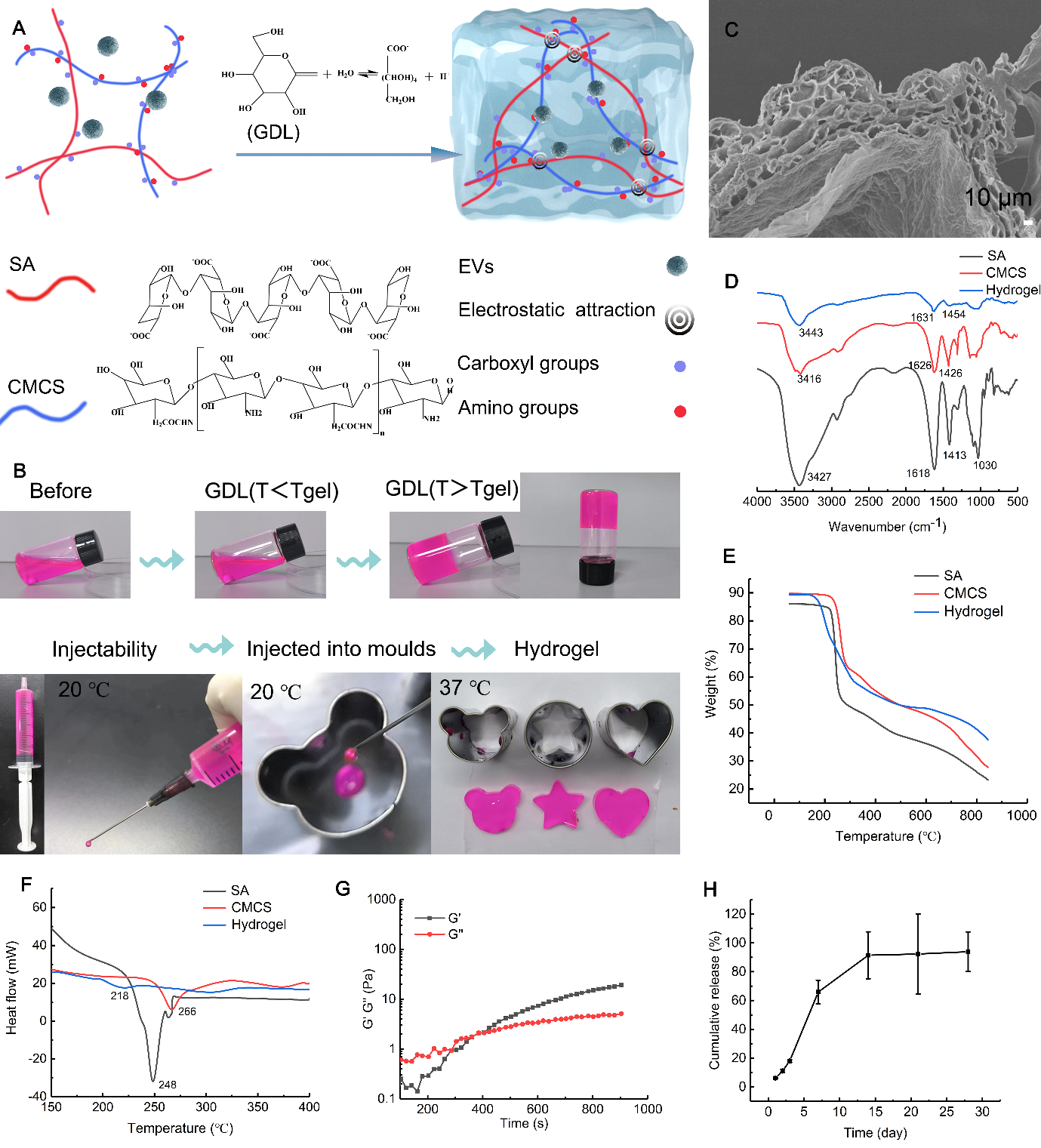


Fig. S5 Characterization of injectable hydrogel. (A) Schematic illustration of designing injectable hydrogel loading exosomes. (B) Photograph of injectable hydrogel with shape adaptability. (C) SEM images of hydrogels. FTIR (D), TG (E), and DSC (F) of the hydrogel after lyophilization. (G) The rheology test of the hydrogel with 2% GDL at 20℃. (H) Cumulative release profile of Milk-Exo from the hydrogel within 28 days. Data are presented as mean ± SD (n = 3).


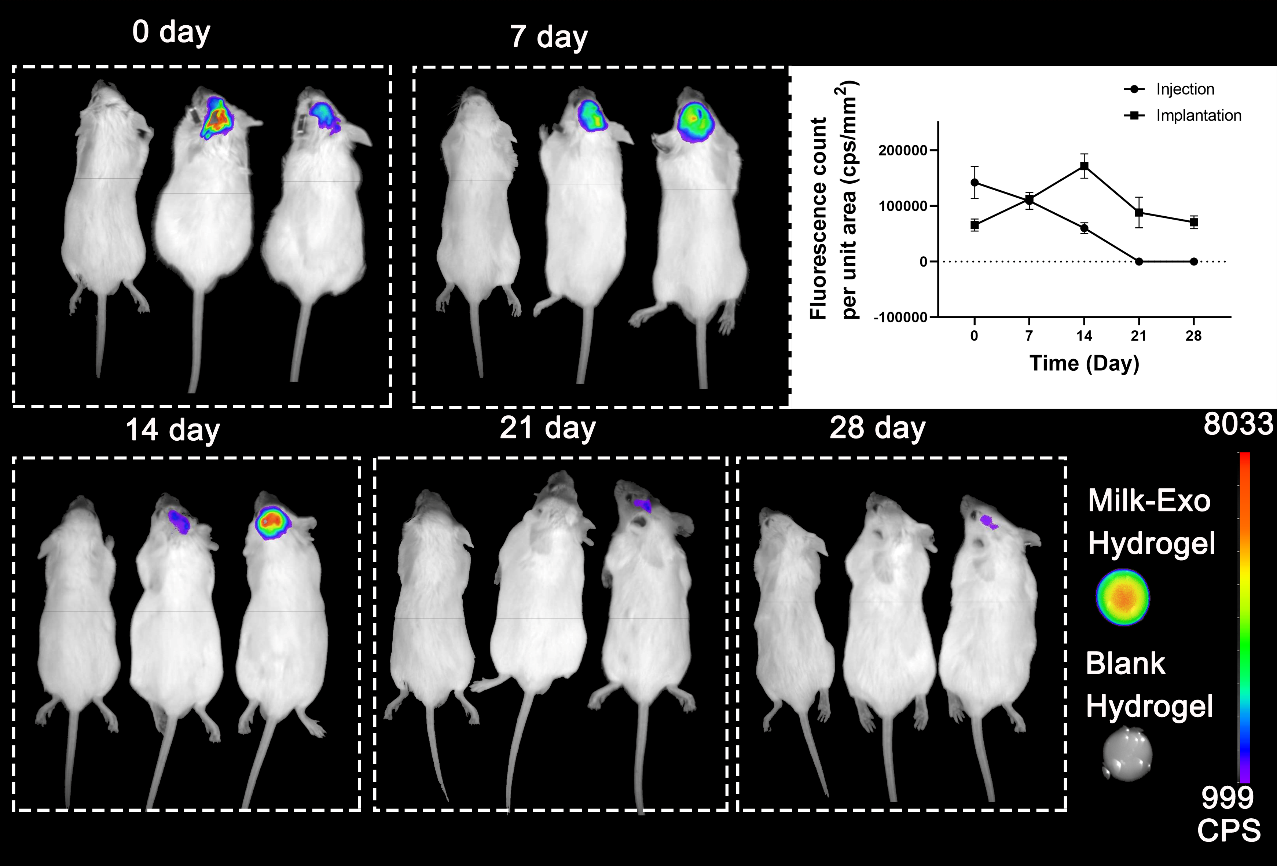


Fig. S6 Fluorescence signals and quantification of control, DiR labeled Milk-Exo injection group, and DiR labeled Milk-Exo@Gel implantation group in TBI mice at day 0, 7, 14, 21, and 28. From left to right are control, DiR labeled Milk-Exo injection, and DiR labeled Milk-Exo@Gel implantation group. Data are presented as mean ± SD (n = 3).


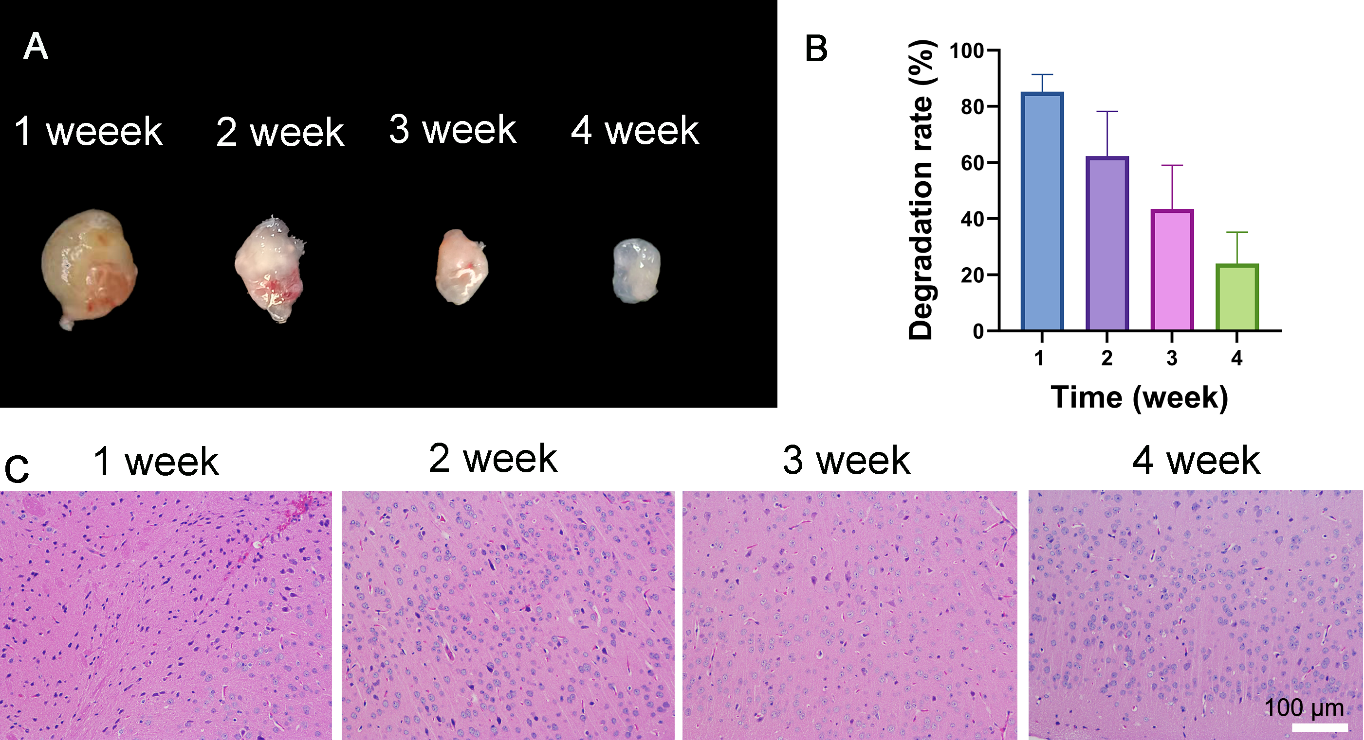


Fig. S7 Degradation behavior of hydrogel. Images (A) and degradation rate (B) of hydrogel at different times. (C) H&E staining of brain tissue in the presence of hydrogel for different times. Data are presented as mean ± SD (n = 3).


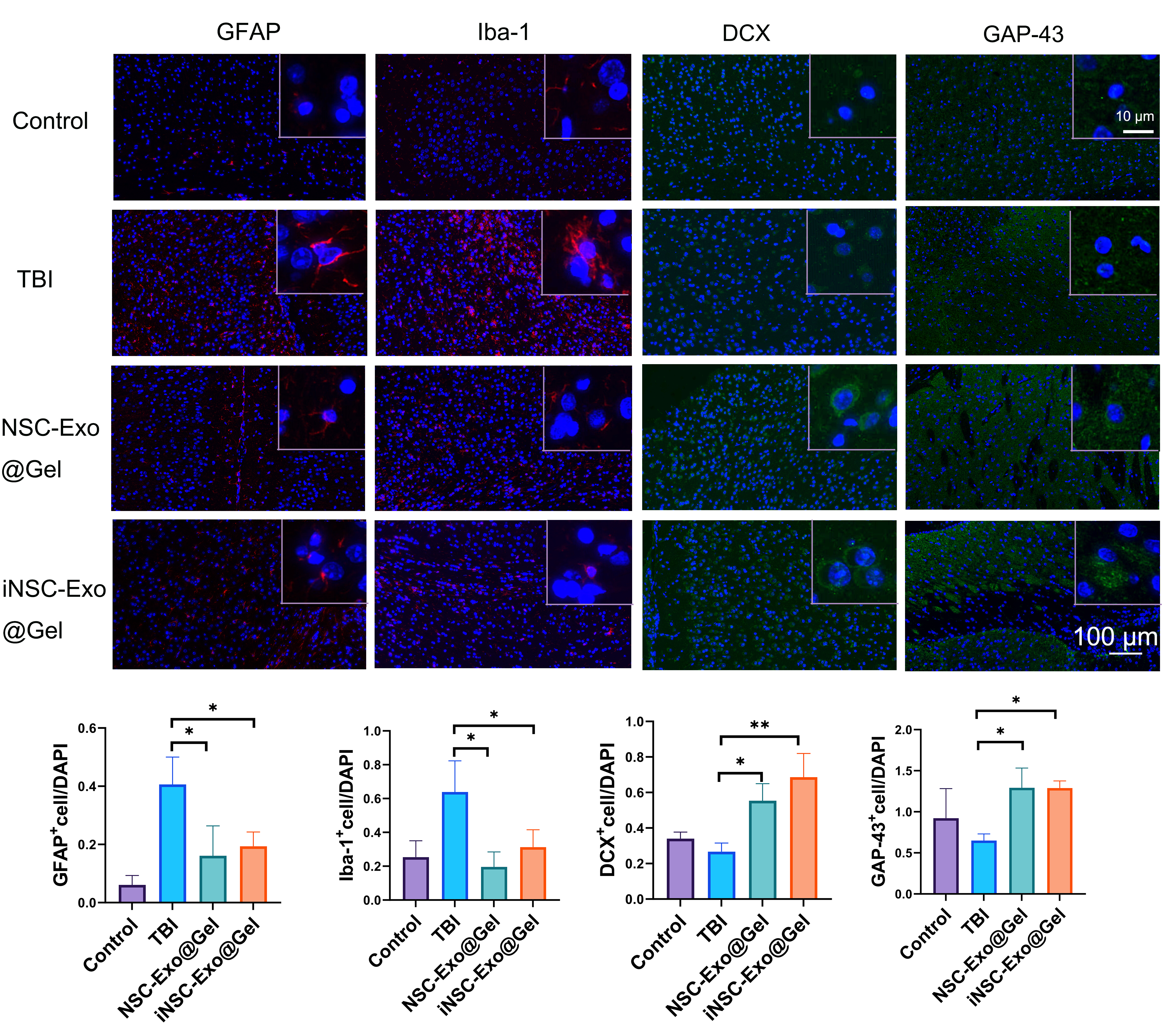


Fig. S8 NSC-Exo@Gel and iNSC-Exo@Gel exerted therapeutic effects on TBI mice by inhibiting neuroinflammation and promoting nerve regeneration. The enlarged image is in the upper right corner. Expression levels of GFAP, Iba-1, DCX, and GAP-43 were quantified in each group on day 28 post-injury. Data presented as mean ± SD, n=3, P-values are calculated using one-way ANOVA with Tukey’s multiple-comparisons test, *P＜0.05, **P＜0.01.


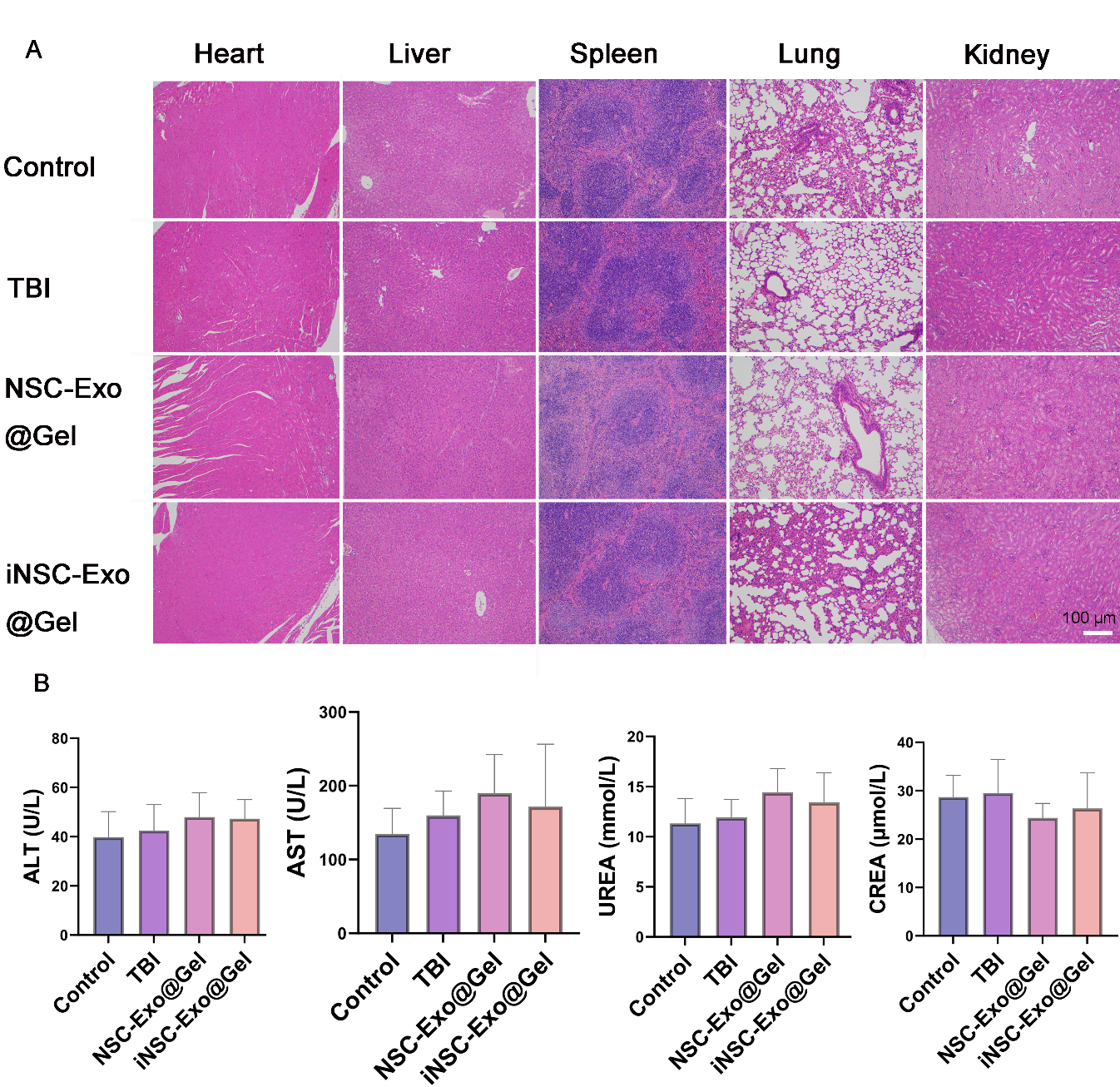


Fig. S9 (A) H&E images of heart, liver, spleen, lung, kidney at 28 days post-surgery. (B) The effect of biochemical test on the function of liver and kidney at 28 days after treatment. Data presented as mean ± SD, n=6, P-values are calculated using one-way ANOVA with Tukey’s multiple-comparisons test.


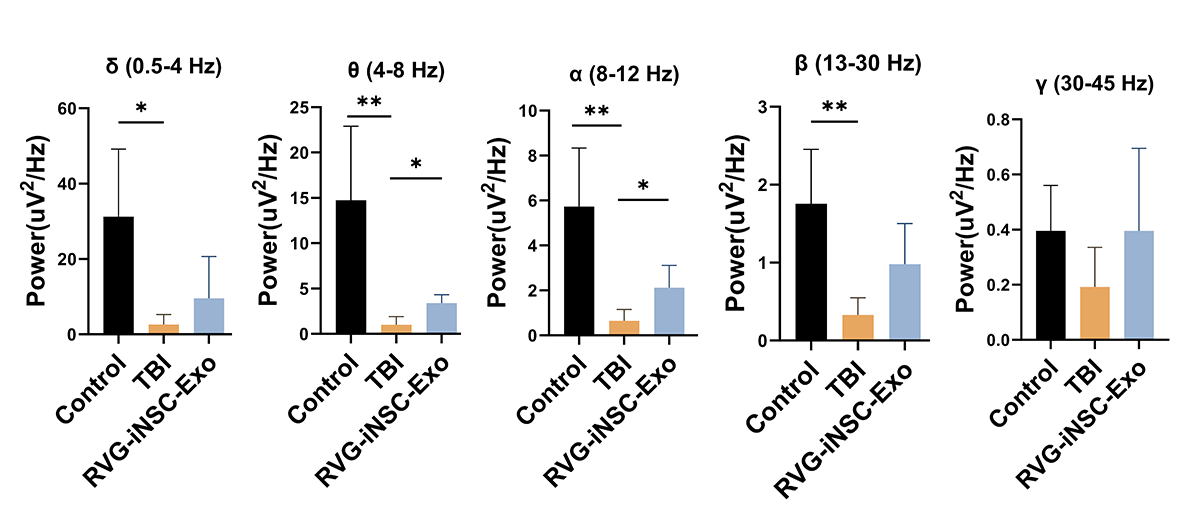


Fig. S10 Quantification of average delta, theta, alpha, beta, and gamma band power in the hippocampus. Data presented as mean ± SD, n=3, P-values are calculated using one-way ANOVA with Tukey’s multiple-comparisons test, *P＜0.05, **P＜0.01.


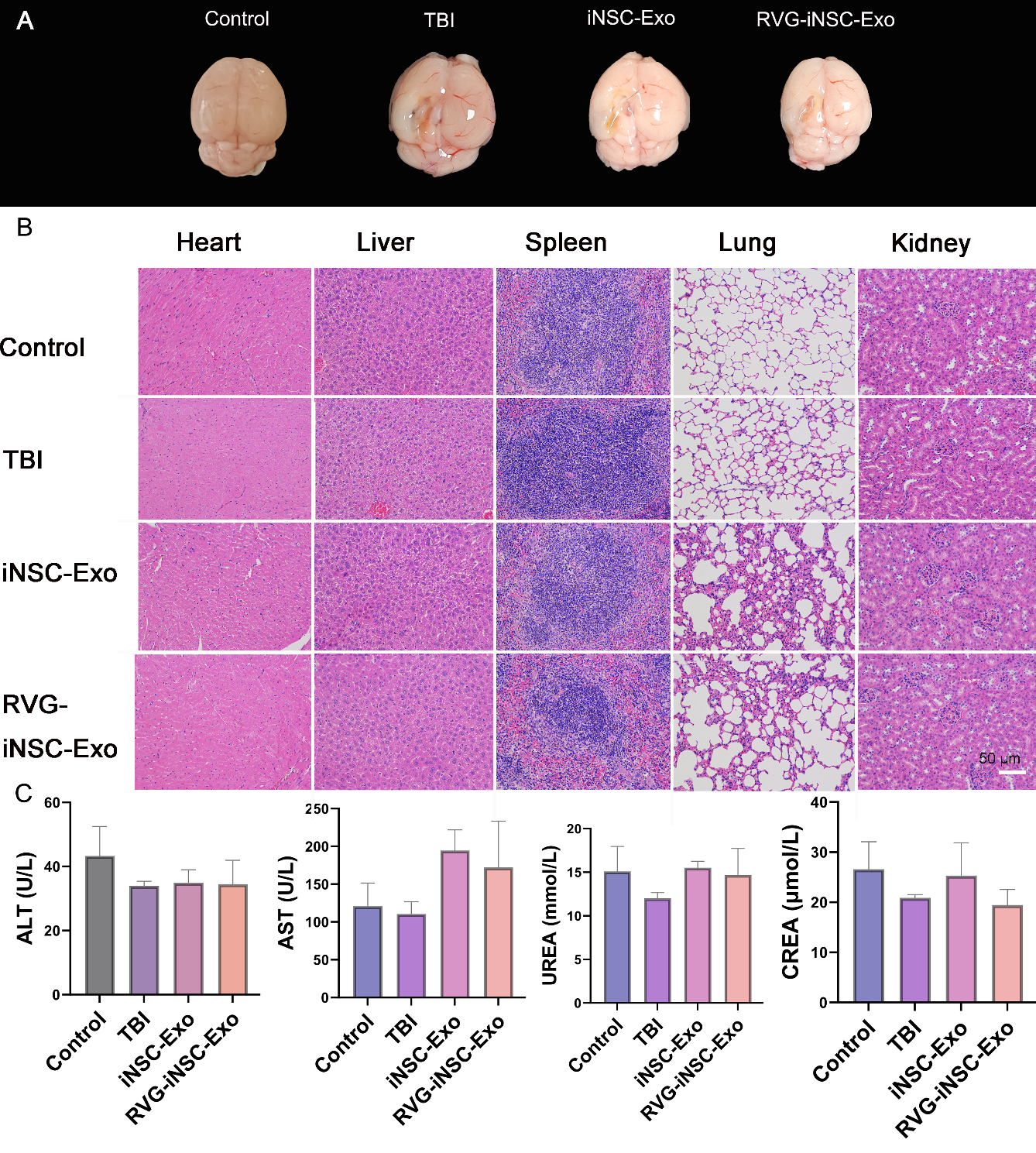


Fig. S11 (A) Representative picture showing the brain defects at 22 days after systemic administration. (B) H&E images of heart, liver, spleen, lung, kidney at 22 days after systemic administration. (C) The effect of biochemical test on the function of liver and kidney at 22 days after treatment. Data presented as mean ± SD, n=3, P-values are calculated using one-way ANOVA with Tukey’s multiple-comparisons test.

Fig. S12 (A) H&E images and representative pictures showing the brain defects at 22 days post-surgery. (B) The trauma area was quantified. Data presented as mean ± SD, n=3, P-values are calculated using one-way ANOVA with Tukey’s multiple-comparisons test, *P＜0.05.


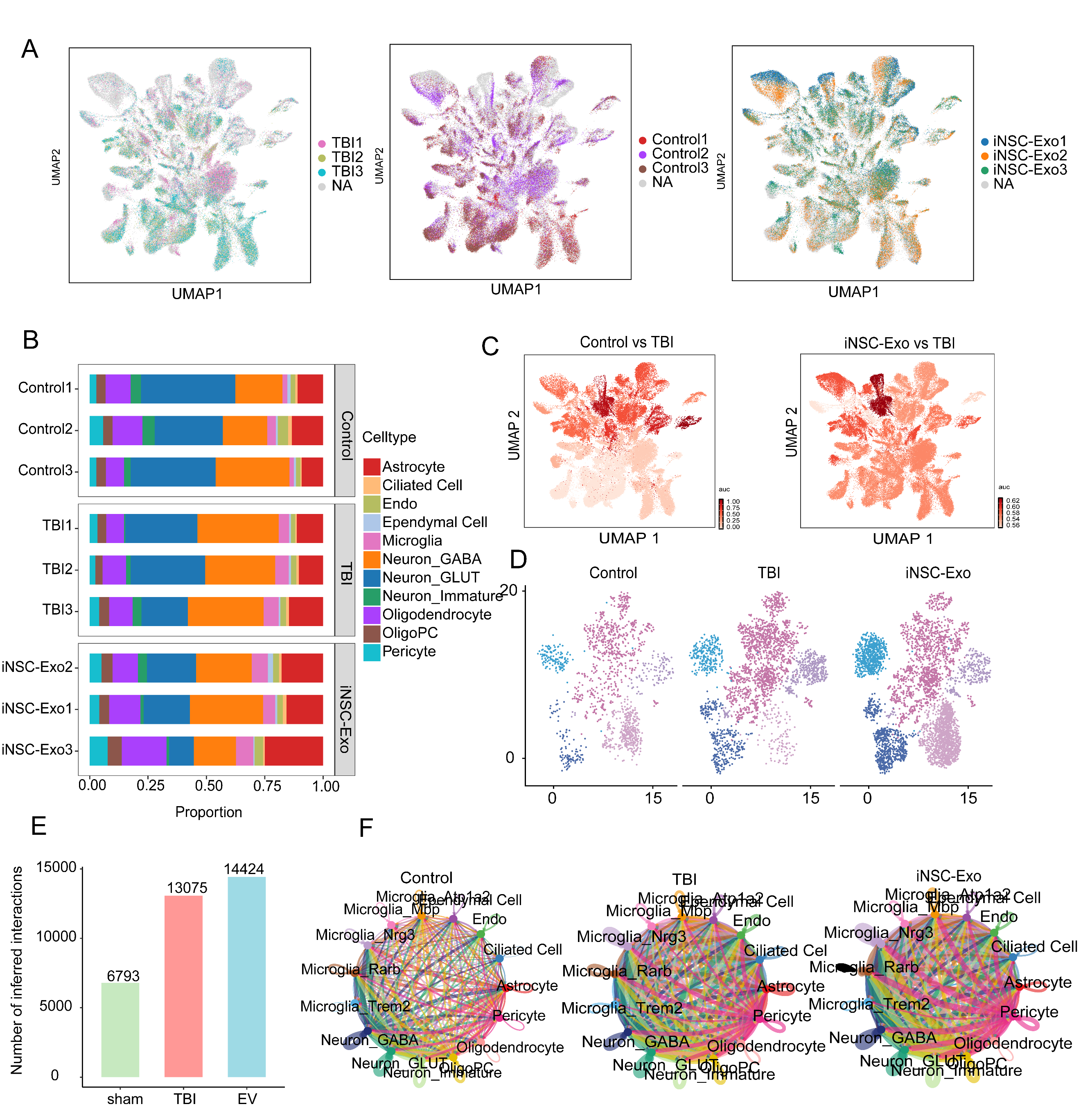


Fig. S13 (A) The visualization of cell classes was achieved using UMAP in three groups among different samples. (B) A bar chart illustrating the distribution of 11 cell types across various samples in three groups. (C) The UMAP plot utilizing Augur demonstrates the cells exhibiting the most significant variability, as determined by AUC values, in association with TBI in comparison to the control group and iNSC-Exo group. (D) The UMAP analysis displays 5 microglia subpopulations among 3 groups. (E) Bar charts illustrating the variations in the number of interactions within the cell-cell communication network among the Control, TBI, and iNSC-Exo groups. (F) Circle charts illustrating the variations in the number of interactions within the cell-cell communication network among the Control, TBI, and iNSC-Exo groups.


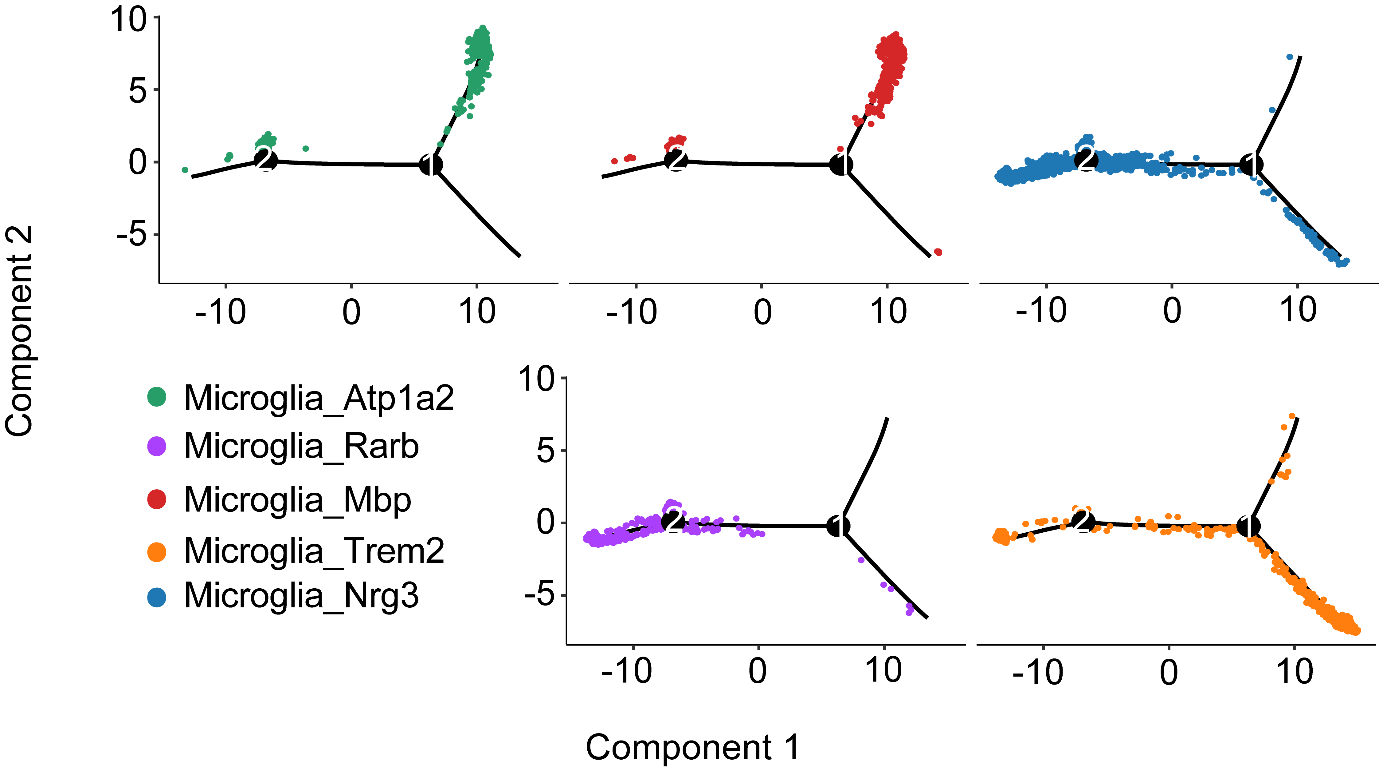


Fig. S14. The prospective developmental pathways of five microglial were inferred using Monocle split by subpopulations.


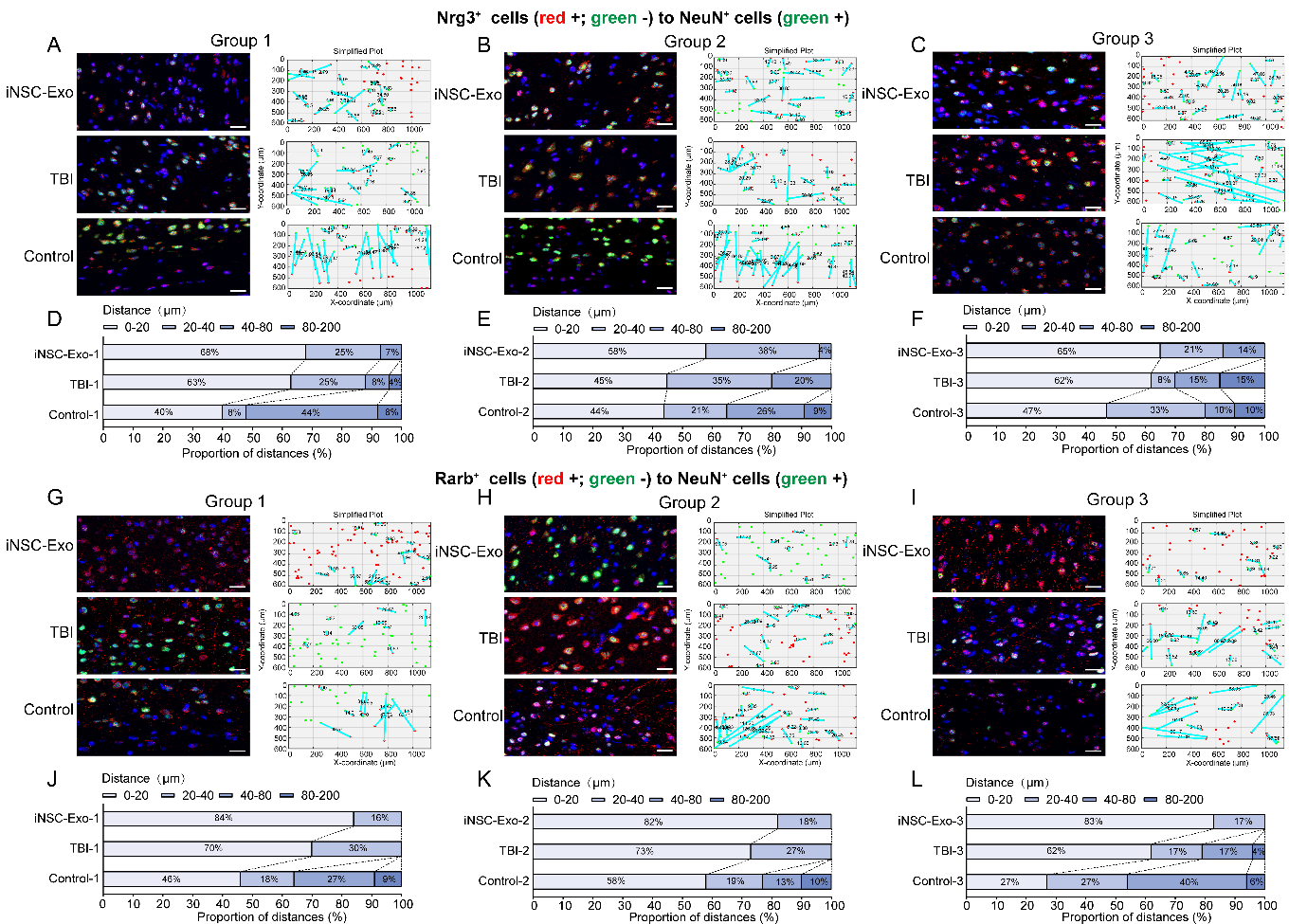


Fig. S15. Multiplex immunohistochemical staining enables quantification of spatial distances between Nrg3+ or Rarb+ non-neuronal cells and NeuN+ neurons in TBI mouse brain tissue. (A-C) Representative immunofluorescence images (left) and corresponding simplified distance plots (right) of parallel samples from the iNSC-Exo, TBI, and Control groups. Nrg3 (red) and NeuN (green) fluorescence are shown. (D-F) Stacked proportion plots quantify the distance distribution between Nrg3+&NeuN− cells and NeuN+ cells across the three groups (n=10-39). (G-I) Immunofluorescence staining (left) and simplified distance plots (right) of parallel samples from the iNSC-Exo, TBI, and Control groups, showing Rarb (red) and NeuN (green) signals. (J-L) Stacked proportion plots analyzing the spatial distribution of Rarb+&NeuN− cells relative to NeuN+ cells in all groups (n=10-39). Scale bar: 20 μm.


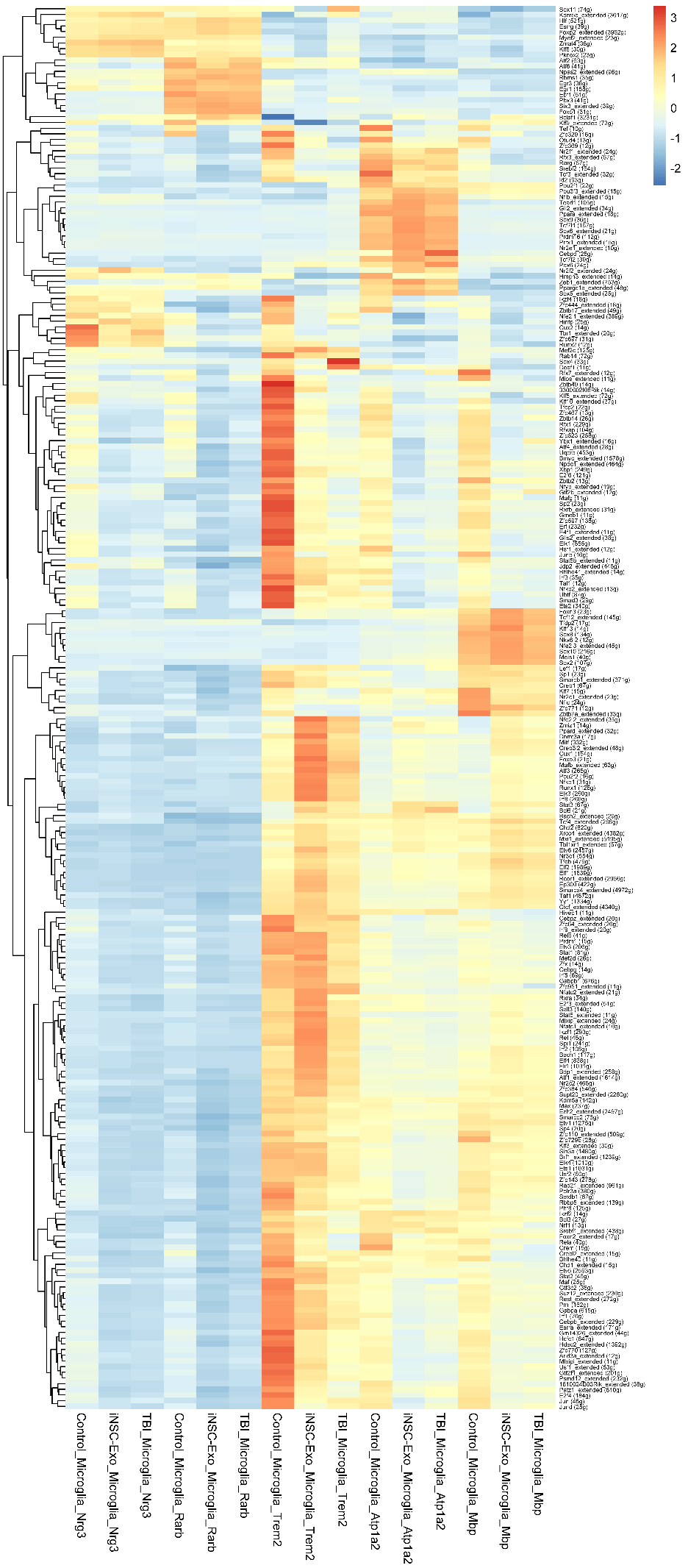


Fig. S16 The heatmap illustrates the cell type-specific regulons as determined by the SCENIC algorithm, with regulon activity levels ranging from “low” (indicated in blue) to “high” (indicated in red).


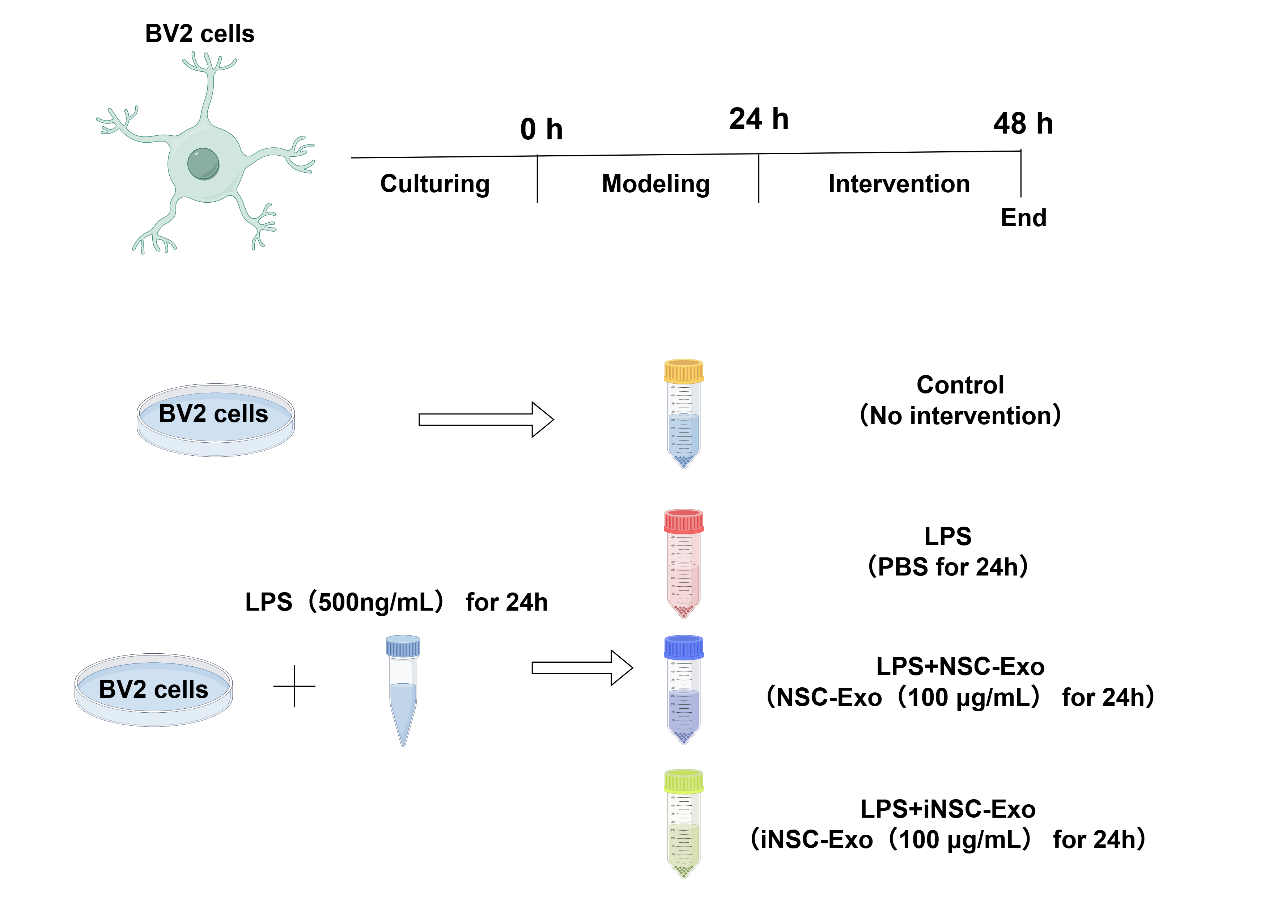


Fig. S17 In vitro BV2 cells experiment timeline and grouping.
